# IFIT3 mediates TBK1 phosphorylation to promote activation of pDCs and exacerbate systemic sclerosis in mice

**DOI:** 10.1002/ctm2.1800

**Published:** 2024-09-20

**Authors:** Xiangyang Huang, Yi Liu, Xia Rong, Yiheng Zhao, Dan Feng, Jun Wang, Wanhong Xing

**Affiliations:** ^1^ Department of Rheumatology and Immunology West China School of Public Health and West China Fourth Hospital, Sichuan University Chengdu China; ^2^ Department of Communication Sciences & Disorders MGH Institute of Health Professions Boston Massachusetts USA; ^3^ Department of Cardiothoracic Surgery The Sixth People's Hospital of Chengdu Chengdu Sichuan China

**Keywords:** CRISPR/Cas9, fibrosis, IFIT3/TBK1 signalling pathway, pDCs activation, scRNA‐seq, systemic sclerosis

## Abstract

**Objective:**

To assess the impact of the IFIT3/TBK1 signalling pathway in activating plasmacytoid dendritic cells (pDCs) and its role in the development of SSc.

**Methods:**

Utilized single‐cell RNA sequencing (scRNA‐seq) and high‐throughput transcriptome RNA sequencing to reveal the differential abundance of pDCs and the role of the key gene IFIT3 in SSc. Conducted in vitro cell experiments to evaluate the effect of IFIT3/TBK1 signalling pathway intervention on pDC activation cytokine release and fibroblast function. Constructed an IFIT3^−/−^ mouse model using clustered regularly interspaced short palindromic repeats (CRISPR)/CRISPR‐associated protein 9 (Cas9) gene editing to assess the potential benefits of intervening in the IFIT3/TBK1 signalling pathway on skin and lung fibrosis in the SSc mouse model.

**Results:**

The IFIT3/TBK1 signalling pathway plays a crucial role in activating pDCs, with IFIT3 acting as an upstream regulator of TBK1. Intervention in the IFIT3/TBK1 signalling pathway can inhibit pDC activation cytokine release and impact fibroblast function. The IFIT3^−/−^ mouse model shows potential benefits of targeting the IFIT3/TBK1 signalling pathway in reducing skin and lung fibrosis in the SSc mouse model.

**Conclusion:**

This study provides new insights into potential therapeutic targets for SSc, highlighting the critical role of the IFIT3/TBK1 signalling pathway in SSc development.

**Highlights:**

- This study elucidates the pivotal role of plasmacytoid dendritic cells (pDCs) in systemic sclerosis (SSc).
- This study identified the key regulatory gene involved in systemic sclerosis (SSc) as IFIT3.
- This study has found that IFIT3 functions as an upstream regulatory factor, activating TBK1.
- This study provides Evidence of the regulatory effects of the IFIT3/TBK1 pathway on plasmacytoid dendritic cells (pDCs).
- This study validated the therapeutic potential using the IFIT3^−/−^ mouse model.

## Graphical abstract

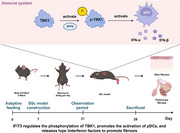

## 1 INTRODUCTION

Systemic sclerosis (SSc) is a multifaceted autoimmune disorder distinguished by pervasive irregularities in small blood vessels, the production of specific antibodies and the fibrosis of multiple organs.^1^, ^2^, ^3^, ^4^, ^5^, ^6^ The occurrence and progression of this disease are influenced by various factors, such as genetic factors, immune abnormalities and environmental factors.^7^ It is noteworthy that immune cells are pivotal in the pathological processes of SSc.^8^, ^9^, ^10^, ^11^ Plasmacytoid dendritic cells (pDCs), as a distinct category of immune cells, play pivotal roles in various autoimmune diseases. The aberrant activation of pDCs is closely linked to the onset and progression of SSc.^12^, ^13^ In the pathological state of SSc, pDCs could contribute to inflammatory response and fibrosis through the secretion of excessive levels of type I interferons (IFNs) and pro‐inflammatory factors.^13^, ^14^, ^15^

IFIT3, also known as Interferon Induced Protein 3, plays a crucial role as an antiviral protein, with its expression being controlled by different IFNs and viral infections.^16^, ^17^ IFIT3 is essential for the proper functioning of the immune response against viral infections and in regulating inflammation in various contexts.^18^ Several recent studies have demonstrated that IFIT3 is also in the development and advancement of various autoimmune diseases.^19^, ^20^, ^21^ For instance, IFIT3 could regulate cellular apoptosis and survival, consequently impacting the function and population of immune cells.^22^ IFIT3 plays a crucial role in modulating multiple signalling pathways, such as the TBK1 signalling pathway. Moreover, IFIT3 is involved in innate immune responses and inflammatory reactions.^23^, ^24^

TANK‐binding kinase 1 (TBK1), as a vital component in multiple physiological functions, particularly in viral infections and immune reactions, holds significant importance. It regulates downstream IFN signalling pathways and influences immune cell function.^25^, ^26^, ^27^ Numerous studies have indicated that the multiple autoimmune conditions are influenced significantly by the TBK1 signalling pathway, impacting their onset and progression.^28^, ^29^, ^30^ TBK1 affects cell survival and apoptosis and could also impact pathological processes by regulating the inflammatory environment, particularly in specific inflammatory diseases and cancers.^31^ The interaction between IFIT3 and TBK1 and their role in immune cell activation regulation, specifically pDCs in SSc, have not been completely explained.^24^

Furthermore, the clustered regularly interspaced short palindromic repeats (CRISPR)/CRISPR‐associated protein 9 (Cas9) gene editing technology has found wide application in various biomedical research fields because of its precise and efficient gene targeting capability.^32^ This technique enables precise deciphering of gene function and its role in disease development and progression through knockout or modification of specific gene expressions.^33^ Previous studies on SSc have successfully used CRISPR/Cas9 technology to reveal the relationship between different genes and the occurrence and development of SSc. It has opened up new avenues for researching related mechanisms.^34^

Considering the association of IFIT3/TBK1 with autoimmune diseases, we propose a potential relevance of IFIT3/TBK1 in SSc. Through the application of single‐cell RNA sequencing (scRNA‐seq) and high‐throughput RNA sequencing analysis, we identified key immune cells, core genes and signalling pathways involved in the occurrence and progression of SSc, with a focus on the IFIT3/TBK1 pathway. By conducting in vitro cellular experiments and utilising gene editing techniques, we aim to elucidate the role of the IFIT3/TBK1 pathway in regulating the functions of pDCs and the pathogenesis of SSc. Our study aims to offer novel molecular targets and therapeutic options for the diagnosis and management of SSc, thereby holding great significance for both scientific research and clinical interventions.

## 2 MATERIALS AND METHODS

### 2.1 Constructing the SSc model

Male C57BL/6J mice, aged 5−6 weeks, were acquired from Beijing Vitectly Hua Experimental Animal Technology Co., Ltd. The mice were bred in laboratories with SPF‐level conditions, including a humidity range of 60–65% and a temperature range of 22–25°C. All mice have unrestricted access to water. Monitor the health status of the mice after 1 week of acclimatisation. The experimental protocol and animal usage plan have received approval from our institution's Animal Ethics Committee.

Initially, the mice were randomly assigned to either the control or model groups, each consisting of three mice (*n* = 5). The mice in the model group underwent intraperitoneal injections of bleomycin daily at a dose of 4 U/kg (9041‐93‐4; Sigma–Aldrich), whereas the mice in the control group received injections of 0.9% NaCl in equivalent amounts. The injection is administered over 2 weeks. After the 28th day, each group of mice is euthanised using cervical dislocation anaesthesia.^35^

### 2.2 scRNA‐seq and data analysis

Lung tissues were collected from both the control and model groups, with three samples selected from each group. A single‐cell suspension was prepared using a pancreatic enzyme (9002‐07‐7; Sigma–Aldrich, USA). Single cells were captured using the 10× Chromium Single Cell Gene Expression Solution (Fluidigm, Inc., USA). Once the cells are captured, they are lysed on the chip, releasing mRNA. Then, perform reverse transcription to generate complementary DNA (cDNA). The cDNA molecules undergo post‐cracking and reverse transcription. Subsequently, they are pre‐amplified within a microfluidic chip to facilitate sequencing. Compilation of libraries was carried out by amplified cDNA, and single‐cell sequencing was executed on the HiSeq 4000 Illumina platform under the following parameters: paired‐end sequencing, featuring a read length of 2 × 75 bp and an average of approximately 20 000 reads per cell.^36^

Utilisation of the ‘Seurat’ package within the R software facilitated the analysis of the data. During the data quality control process, the filtering criteria employed are 200 < nFeature_RNA < 5000 and percent.mt < 20. Then, select 2000 genes with highly variable expression.^37^

To decrease the complexity of the scRNA‐Seq dataset, a principal component analysis (PCA) was conducted based on the variances of 2000 genes that exhibited significant variability. Conduct downstream analysis using the top 20 principal components (PCs) selected through the Elbowplot function from the Seurat package. Identify the main cell subpopulations using the FindClusters function provided by Seurat, with the resolution set to the default value (res = 1). Next, apply the t‐SNE algorithm for nonlinear dimensionality reduction on scRNA‐seq data. The Seurat package should filter marker genes for different cell subtypes and annotate marker genes specific to individual cell clusters through the utilisation of the ‘SingleR’ package.^38^ Last, conduct cell communication analysis utilising the ‘CellChat’ software package in the R programming language.^39^

### 2.3 Transcriptome high‐throughput sequencing and data analysis

Lung tissues were collected from a control group (*n* = 3) and a model group (*n* = 3) and promptly stored in RNAlater storage solution (76104; Qiagen) to preserve RNA stability. Performing transcriptome high‐throughput sequencing analysis using RNA sequencing. The following steps were performed: the isolation of total RNA from each sample was achieved through the application of Trizol reagent (T9424; Sigma–Aldrich) following the manufacturer's specified guidelines. Ultraviolet–visible spectrophotometry (model: 840−343700; Thermo Scientific) could be employed to determine the quality and concentration of RNA. The acceptable range for the A260/280 ratio is 1.8 to 2.0. Each sample contains a total RNA content of 3 µg, employed as the foundational material for RNA sample construction. Abiding by the directives specified by the manufacturer, the cDNA libraries were prepared through the NEBNext® UltraTM II RNA Library Prep Kit for Illumina (E7770S; Gene Company, China). Evaluation of library quality was conducted through the application of an Agilent Bioanalyzer 2100 system. The indexed samples should be clustered on the cBot Cluster Generation System using the TruSeq PE Cluster Kit v3 cBot HS (Illumina), complying with the directives outlined by the manufacturer. Following the clustering process, the libraries underwent sequencing using the Illumina‐HiSeq 550 platform, which produced paired‐end reads with lengths of 125/150 bp.^40^

After acquiring the gene expression matrix, conduct differential analysis utilising the limma package in R software. Differentially expressed genes (DEGs) in lung tissue between the control and model groups were identified using a threshold of |logFC| > 2 and *p* < 0.05. Generate a volcano plot to display variances in gene expression using R software's ‘pheatmap’ package.

### 2.4 Wayne analysis

We retrieved key genes using the keywords ‘Systemic sclerosis’ and ‘Interstitial Pulmonary Fibrosis’ from the DisGENET database (https://www.disgenet.org/). After sorting the data and removing duplicate values, utilise the ‘Draw Venn Diagram’ tool for Venn analysis on the DEGs obtained from both pDC and transcriptional profiling. It will allow the identification of the intersecting genes.^41^

### 2.5 Cell culture and treatment

Flow cytometry isolated pDCs from the spleens of normal C57BL/6J mice. The mouse fibroblast cell line MEF (BFN60808719) was procured from BLUEFBIO (Shanghai, China). Utilising Dulbecco's modified Eagle medium (DMEM) (Thermo Fisher; prod. no. 11965092), 1% penicillin–streptomycin (Gibco; prod. no. 10378016) and 5% foetal bovine serum (FBS) (Gibco; prod. no. 10099141C), the cells were cultured. Subsequently, the cell cultures were maintained in a 37°C incubation chamber supplemented with 5% CO_2_ for optimal growth conditions and the medium was replaced every 7 days.^36^

Transfected pDCs expressing IFIT3 or TBK1 were seeded onto a 48‐well plate with 2 × 10^5^ fibroblasts for co‐culture. RPMI‐1640 medium (SLM‐240; Sigma–Aldrich) was used to culture the cells, along with 1% penicillin–streptomycin (10378016; Gibco), 2 mM l‐glutamine, 1 mM sodium pyruvate, 1× MEM non‐essential amino acids and 10% FBS as supplements.^42^, ^43^

### 2.6 Cell grouping and transfection

Gene‐silenced or overexpressed cell lines could be created using the lentivirus transduction method and corresponding control cell lines. Simultaneously, knock down two groups of shRNA sequences and select the more effective group for further experiments. Silent viral sequences and their effects are presented in Table 1 and Figure S1. The Phage‐puro series plasmids overexpressing IFIT3 or TBK1 were transfected into pDCs in addition to the helper plasmids Pspax2 and Pmd2.G. Similarly, the pSuper‐retro‐puro series plasmids containing silencing sequences targeting IFIT3 or TBK1 were transfected into pDCs along with the helper plasmids gag/pol and VSVG. Sangon Biotech (Shanghai) Co., Ltd. offers plasmid and lentivirus packaging services. The experiments involved the division of pDCs into the subsequent groups: (1) sh‐NC, sh‐IFIT3, oe‐NC and oe‐IFIT3; (2) sh‐NC, sh‐TBK1, oe‐NC and oe‐TBK1; (3) sh‐NC+oe‐NC, sh‐IFIT3+oe‐NC, sh‐IFIT3+oe‐TBK1, oe‐IFIT3+sh‐NC and oe‐IFIT3+sh‐TBK1.

**TABLE 1:** Lentivirus transfection sequence.

Name	Sequence(5′−3′)
sh‐NC	TTCTCCGAACGTGTCACGT
sh‐IFIT3‐1(Mouse)	GCCTACATAAAGCACCTAGAT
sh‐IFIT3‐2(Mouse)	GCGAAGTCCTTTGAACTCCTA
sh‐TBK1‐1(Mouse)	GCTGGCCGAGAACAATCATAT
sh‐TBK1‐2(Mouse)	CCAGAATCAGAATTTCTCATT
IFIT3(Mouse)	F: ATGAGTGAGGTCAACCGGGAAT
	R: CTATGTTTGCTCTTTAACCTCT

To mediate the transfection of cells using lentiviral vectors, six‐well plate was loaded with 1 × 10^5^ cells. Once the cells reached a confluence of 70−90%, the medium was supplemented with a suitable concentration of packaged lentiviral vectors (MOI = 10, working titre approximately 1 × 10^6^ TU/mL) and 5 µg/mL polybrene (TR‐1003; Sigma–Aldrich) to facilitate the transfection process. After 4 h, the polybrene was diluted by adding an equal medium volume. Subsequently, the medium was refreshed following a 24‐h interval. Opting for persistent cell lines, 1 µg/mL puromycin (A1113803; Thermo Fisher) was introduced after 48 h of transfection to eradicate the non‐resistant cells.^44^, ^45^

### 2.7 CCK‐8, colony formation and cell scratch experiments

The CCK‐8 assay kit (CK04; Dojindo Laboratories) was employed for the evaluation of cell viability. Inoculation of 1 × 10^5^ cells was performed in every well of a 96‐well plate. Then, the procedure involved the addition of 10 µL of CCK‐8 solution to each well per day, followed by the addition of 100 µL of serum‐free culture medium. The plate underwent incubation at 37°C for 2 h, following which the optical density at 450 nm was assessed.

During the settlement formation experiment, cultures were initiated with 100 000 cells per well and nurtured for about 2 weeks in a medium supplemented with 10% FBS, with medium changes performed every 5 days. After 14 days, the bacterial colonies were treated with methanol as a fixative and then stained using a 0.1% crystal violet solution (548‐62‐9; MCE). The formation rate of colonies is determined by counting the number of colonies stained using cell counting after 14 days in each group.^46^

In the scratch healing experiment, cells were cultured at 100% density in a six‐well plate using a serum‐free medium. Subsequently, a disruption was introduced to the cell monolayer by utilising a 200 µL pipette tip, and representative images of cell migration were captured 24 h later. The width of the wound bed was measured and recorded. The distance between the damaged area and the control area at 0 h was measured and calculated using the relative migration rate^47^ to analyse the extent of the induced damage.

### 2.8 Transwell detection of cell migration

The experiment utilised 24‐well plates with 8.0 µm Transwell (3464; Corning) and Matrigel (354234; Corning) coatings for migration and invasion assays. Cultured fibroblast cells were placed in the upper well of the Transwell system at a concentration of 1 × 10^5^ cells/well and 5% FBS was added. Transfected pDCs were plated onto the lower compartment of the Transwell at a concentration of 1 × 10^5^ cells per well and 10% FBS was added. The migration experiment was conducted post‐incubation at 37°C for a duration of 24 h. Next, the HeLa cells were fixed with 100% methanol for 20 min and stained with 0.1% crystal violet for 20 min. The cell count in every group should be examined using an optical microscope.^48^, ^49^

### 2.9 Enzyme‐linked immunosorbent assay experiment

Expression levels of IFN‐α (221001; ThermoFisher), IFN‐β (ab252363; Abcam), collagen Iα1 (ab210579; Abcam) and IL‐6 (ab222503; Abcam) were quantified in peripheral blood, tissue or cell culture supernatants using enzyme‐linked immunosorbent assay (ELISA) kits. To begin, dilute the antigens in a diluent solution to the appropriate concentration. Then, add the diluted samples to the wells of an ELISA plate. Next, introduce the enzyme‐labelled antibody and substrate solution. Last, cease the reaction by introducing 50 µL of stop solution to every well and measure the experimental results within a 20‐min timeframe. The enzyme label reader (Bio‐Rad, USA; model 1681135) was employed to analyse the plate under 450 nm conditions, and the standard curve was drawn to analyse the data.^42^ The tests mentioned above are conducted obeying the guidelines set forth in the manual supplied by the manufacturer. To ensure the results’ accuracy, conducting a minimum of three tests on each sample is recommended.

### 2.10 Flow cytometry

Tissue digestion was conducted on lung, skin and spleen samples obtained from different groups of mice. The samples were treated with 5 mL of collagenase (17018029; Sigma–Aldrich) at a concentration of 2 mg/mL and then incubated at 37°C for 1 h. The cells were filtered using a 70 µm mesh filter, then centrifuged at 850×*g* for 10 min. After that, the cell samples underwent triple PBS washes and were subsequently resuspended in Percoll (Merck, USA; P1644) as directed by the manufacturer to remove dead cells. The cell concentration needs to be set at 1 × 10^7^ cells/mL before being resuspended in 50 µL of chilled PBS with 2% FBS. To label pDCs, employ CD123 (11‐1231‐82; ThermoFisher) and CLEC4C (ab312789; Abcam) antibodies. Utilise CD3 (ab16669; Abcam) and CD4 (92599; CST) antibodies to label CD4+ T cells. To detect CD8+ T cells, employ CD3 and CD8 (ab237367; Abcam) antibodies. Subsequently, the flow cytometer (LSR II; BD Biosciences) was utilised for sorting, followed by data analysis with the FlowJo software.^50^

### 2.11 RT‐qPCR

Total RNA was obtained from the cells in different groups utilising the Trizol reagent kit (T9424; Sigma–Aldrich, Germany). The determination of RNA quality and concentration was conducted using ultraviolet–visible spectrophotometry (ND‐1000; Nanodrop, USA). The PrimeScript™ RT Kit (RR014B; TaKaRa, Japan) performed reverse transcription and detected the mRNA expression level. Real‐time quantitative reverse transcription polymerase chain reaction (RT‐qPCR) was performed via the TB Green® Premix Ex TaqTM Kit (RR420W; TaKaRa) on the ABI 7500 PCR System (Applied Biosystems, USA). GAPDH is the reference gene, an internal control for mRNA expression. The following are the primer sequences: IFIT3 (forward: 5′‐CCTGTGTACCACAAGGGAACT‐3′, reverse: 5′‐CTGGGGCCACACGAAAGAAA‐3′) and GAPDH (forward: 5′‐AAGAGGGATGCTGCCCTTAC‐3′, reverse: 5′‐GTTCACACCGACCTTCACCA‐3′). Relative quantification was conducted by comparing Ct values using the 2^−ΔΔCt^ method. Here, ΔΔCt is used to illustrate the difference in ΔCt values between the experimental group and the control group, with ΔCt specifying the divergence in Ct values between the target gene and the reference gene.^37^

### 2.12 Western blot

Utilising the protein extraction kit BC3710 from Solarbio, China, proteins were extracted from both cell cultures and tissue specimens. The specimens were subjected to centrifugation at 18,888 *g* and 4°C for 15 min. The supernatant was retrieved, and the protein concentration was evaluated by means of the BCA protein quantification kit (P0010; Beyotime, China). The protein was segregated through SDS‐PAGE electrophoresis and then translocated to a PVDF membrane. The membrane was subsequently obstructed using 5% skim milk for 60 min at ambient temperature. Diluted primary antibodies were applied separately, including: IFIT3 (PA5‐22230, 1/2000; ThermoFisher), TBK1 (67211‐1‐Ig, 1/1000; proteintech), pTBK1 (5483; CST), IFN‐α (PA5‐86767, 1/1000; ThermoFisher), EGFR (ab52894, 1/1000; Abcam), CTGF (ab6992, 1/1000; Abcam), TGF‐β (ab215715, 1/1000; Abcam), α‐SMA (ab5694, 1/1000; Abcam), collagen I (ab138492, 1/1000; Abcam), fibronectin (ab2413, 1/5000; Abcam), tubulin (ab7291, 1/1000; Abcam) and GAPDH (ab8245, 1/5000; Abcam). The samples were then incubated overnight. The membrane needs to undergo triple washing with TBST for 5 min on each occasion. Next, the secondary antibody, either anti‐rabbit‐IgG (7074, 1/1000; CST) or anti‐mouse‐IgG (7076, 1/1000; CST), should be added and the incubation should be carried out at room temperature for 1 h. Post this step, the membrane should undergo a triple wash with TBST for 5 min on each occasion. TBST was eliminated, and a suitable quantity of ECL operational solution (WBULS0500; EMD Millipore, USA) was formulated. The PVDF membrane was soaked in the ECL developing solution and incubated at room temperature for 1 min. The surplus ECL working solution was eradicated from the PVDF membrane, after which the membrane was sealed using plastic wrap and positioned in a dark enclosure for 5−10 min to facilitate development and fixation. Quantification of the grey intensity of each band in Western blot images was conducted through ImageJ analysis software, with GAPDH utilised as the internal reference point.^51^

### 2.13 Ch‐IP experiment

Conduct the chromatin immunoprecipitation (ChIP) assay using the SimpleChIP@ Plus kit complying with the guidance detailed in the kit manual. Initially, crosslink the cells using a 1% formaldehyde solution, subsequently halting the reaction with 0.1 M glycine. Next, the samples underwent pulse ultrasonic treatment to fragment the DNA into fragments with a length of 500−1000 bp. For the primary antibody, IFIT3 (15201‐1‐AP from proteintech) was used at a dilution of 1/500. A negative control, IgG (30000‐0‐AP from proteintech), was used at a dilution of 1/100. As for the positive control, histone H3 (ab1791 from Abcam) was utilised. The incubation was carried out overnight at 4°C. The PCR amplification was conducted using the TBK1 primers (forward: 5′‐TGCTGGGGTTTTGACCAGTT‐3′, reverse: 5′‐TCTTATGCGCCGTCATGTGT‐3′). The ChIP‐PCR samples underwent separation on a 1% agarose gel and were treated with ethidium bromide for the purpose of detecting the DNA fragments that were immunoprecipitated.^52^

### 2.14 Detection of the regulatory relationship between IFIT3 and TBK1 by immunoprecipitation

Overexpression cell lines and corresponding control cell lines were constructed using lentiviral transfection. IFIT3 or TBK1 overexpression plasmids from the Phage‐puro series were co‐transfected with helper plasmids Pspax2 and Pmd2.G into pDCs. Geneworks Bioengineering from Shanghai, China, supplied the plasmids and lentivirus packaging services. Transfected pDCs and normal pDCs were lysed in BC100 lysis buffer (20 mM Tris–HCl pH 7.3, 100 mM NaCl, 10% glycerol, 0.2 mM EDTA, 0.2% Triton X‐100 and protease inhibitors) for the detection of IFIT3 and TBK1 protein interactions. A segment of the passage was retained as an input, while the remaining extract was incubated with Flag‐tagged IFIT3 antibody (1:50, ab118045; Abcam, USA) or His‐tagged TBK1 antibody (1:30, ab40676; Abcam) at 4°C for 1 h. A/G Plus‐Agarose beads (Santa Cruz Biotechnology) were incorporated and left to incubate overnight at 4°C. After stringent washing, bound proteins were dissociated using SDS sample buffer and analysed by Western blot methodology.

### 2.15 Cell contraction detection

Initially, adjust the cell density of the co‐culture system to 2 × 10^6^ cells/mL. Subsequently, add the following components into distinct tubes: 20 µL of minimal essential medium (M0894; Sigma–Aldrich), 10 µL of sodium bicarbonate (25080094; Gibco), 150 µL of PureCol™ EZ gel (5074; Sigma–Aldrich) and 90 µL of the cell suspension from the co‐culture system. Mix the suspension gently and add 250 µL to each well of a 48‐well plate. Incubation under standard culture conditions for 1 h is necessary for solidification. After that, add 750 µL of DMEM medium (15140148; Gibco) containing 1% penicillin/streptomycin (21041025; Gibco) to each well for culturing. On the second day, the gel blocks were removed from the edges of the holes by gently prying them with a blunt 27‐gauge needle. The images were subsequently analysed using ImageJ to assess the change in cell contraction area between 0 and 5 h for each group.^53^

### 2.16 CRISPR/Cas9 gene editing technology

The CRISPR/Cas9 system, comprising gRNA‐T2 and hCas9, should be utilised to knock out the IFIT3 gene. Optimisation of hCas9 functionality has been achieved, with Synthego (USA) furnishing the IFIT3‐gRNA design. The sequence is GGAAGACAGGGUGUGCAACC. The Cas9 vector was digested using the *Xba*I enzyme and purified the resulting product. In vitro synthesis of Cas9 mRNA was obtained through the mMESSAGE mMACHINE™ T7 Ultra Transcription Kit (AM1345; Invitrogen), followed by purification through alcohol precipitation and concentration using the MEGAclear™ Kit (AM1908; Invitrogen).^54^, ^55^

### 2.17 Microinjection and gene typing

Procure female and male C57BL/6J mice, aged 6−8 weeks and weighing 25 ± 2 g, from Beijing Vital River Laboratory Animal Technology Co., Ltd. House them together in a cage for breeding. The mice were kept in SPF‐grade animal facilities with a humidity of 60–65% and a temperature ranging from 22 to 25°C. Unrestricted food and water were made available to the mice throughout the duration of the experiment. The embryos underwent cultivation in vitro at 37°C in an environment with 5% CO_2_ and 95% humidity. For the embryo injection, a sgRNA (at a concentration of 20 ng/mL), Cas9 (at a concentration of 200 ng/mL) and plasmid vector (at a concentration of 10 ng/mL) were used in a DNase‐free microinjection buffer. The buffer consisted of 1 mM Tris and 0.25 mM EDTA and had a pH of 7.5. Transfer the injected embryos into the oviduct of the pseudopregnant mice.

DNA was extracted from mouse tail tissue using the PureLink™ Genomic DNA Extraction Kit (K182000; Invitrogen), following the instructions provided by the manufacturer. PCR amplification was carried out applying 150 ng of genomic DNA. Genotyping of the gene was conducted with Phusion™ high‐fidelity DNA polymerase (F530S; ThermoFisher) and IFIT3 primers (IFIT3‐F: 5′‐CCTGTGTACCACAAGGGAACT‐3′; IFIT3‐R: 5′‐CTGGGGCCACACGAAAGAAA‐3′).^54^

### 2.18 Animal grouping and treatment

The experiment randomly allocated male C57BL/6J mice into the following groups (*n* = 5): (1) control group, model group, model+anti‐PDCA1 group; (2) control+IFIT3 knockout group, model+IFIT3 knockout group; (3) model+IFIT3 knockout+oe‐NC group, model+IFIT3 knockout+oe‐TBK1 group. The genotypes of the transfected mice were identified using genotyping methods. The genotyping procedures followed previously published protocols, utilising primers specific to IFIT3 and TBK1 as outlined in Table 1.

As stated in the reference of the SSc model,^35^ the groups of mice were intraperitoneally injected with 4 U/kg of bleomycin (Sigma–Aldrich; 9041‐93‐4) or an equal amount of 0.9% NaCl saline solution (control group) for 2 weeks. In mice treated with PDCA1 antibody, the injection of 100 µg of anti‐PDCA1 (130‐092‐550; Miltenyi Biotec) into the peritoneal cavity was performed 1 day before and 14 days before the bleomycin injection. Mice were injected with lentivirus via the tail vein. Each group received an injection of 20 µL of either oe‐TBK1 or oe‐NC lentivirus (1 × 10^7^ IU) at a flow rate of 1 µL/min. Examine the transfection efficiency through the Western blot experiment.^56^ Subsequently, on the 28th day, the mice in the respective groups were euthanised while under anaesthesia. Skin and lung tissues were then collected.

### 2.19 Organ pathology examination

The lung tissue was inflated and subsequently immersed in fresh 4% neutral buffered formalin for 24 h at room temperature. Afterward, it underwent gradient alcohol dehydration, transparency and routine paraffin embedding. Sections measuring 5 µm in thickness were consecutively sliced using a paraffin slicing machine. Subsequently, they underwent 1‐h baking at 60°C and were then dewaxed with xylene. Following hydration, H&E staining (C0105S; Beyotime) and Masson trichrome staining (G1340; Solabio) were conducted to make observations.

The tissue slices were initially submerged in a safranin staining solution for 3 min. Subsequently, they were rinsed with distilled water for 10 s and differentiated with a 1% hydrochloric acid ethanol solution for 10 s. The tissue was rinsed with distilled water for 1 min, followed by staining with eosin for 1 min. Subsequently, a brief rinse with distilled water for 10 s was performed. The tissue was then dehydrated using a series of alcohol gradients and clarified with xylene before being mounted using neutral gum for sectioning.^57^, ^58^

The slices should be immersed in Bouin's solution for 15 min, then rinsed with distilled water for 5 min. Then, the slices should be stained with Weigert's haematoxylin for 5 min and rinsed with distilled water for 5 min. Subsequently, stain the slices with Biebrich scarlet‐acid fuchsin dye for 5 min and proceed to rinse them in distilled water for the same duration. Afterward, the slices should be immersed in phosphotungstic‐phosphomolybdic acid and aniline blue dyes for 5 min each, fixed in 1% acetic acid for 2 min and briefly rinsed with distilled water for 5 min. Gradually dehydrate the slices in alcohol, clear them in xylene and finally mount them on slides using neutral gum.^59^

Morphological changes in lung and skin tissue were visually examined using an optical microscope (XP‐330; Shanghai Bingyu Optical Instrument Co., Ltd., Shanghai, China) following the sectioning process.

### 2.20 Immunohistochemical staining

The embedded tissues should be cut into sections that are 5 µm thick. Subsequently, immunohistochemical staining should be performed following the standard protocol after dehydration. Treat the sample with 3% hydrogen peroxide at ambient conditions for a duration of 10 min to obstruct endogenous peroxidases. After that, add normal goat serum and block for another 10 min. Specific primary antibodies against collagen IV (ab6586, 1:200; Abcam), EGFR (ab52894, 1/100; Abcam) and CTGF (ab6992, 1:200; Abcam) were used. Antibodies were diluted in 0.3% Triton‐X (9036‐19‐5; Sigma–Aldrich) and 2% normal serum and the samples were incubated overnight at 4°C. Following that, wash the sample three times with PBS. Subsequently, incubate it with anti‐rabbit‐HRP (12‐348, 1:1000; Sigma–Aldrich) for 30 min. Then, use the DAB staining kit (ab64238; Abcam) and examine the resulting stain under an optical microscope. Once the colour development is complete, capture images under the microscope. Each group consisted of five animals. Each animal was dyed using one slice. Only one field of view was selected for imaging. The proportion of positive area was calculated using ImageJ software (NIH).^37^

### 2.21 Statistical analysis

The cell clusters obtained from single‐cell sequencing were automatically annotated using the Bioconductor/R software package ‘SingleR’, identifying various cell types including monocytes, T‐cells, neutrophils, natural killer cells, macrophages, pDCs, conventional dendritic cells, myofibroblasts and B‐cells. GraphPad Prism 8.0 was utilised for visualising other data. The data are represented as mean ± standard deviation (mean ± SD). The experimental data encompassed high‐throughput sequencing analysis and in vitro and in vivo experimental results. The comparison between two groups was carried out using non‐paired *t*‐tests, and for comparisons among multiple groups, one‐way analysis of variance was applied. Homogeneity of variance was assessed using the Levene test. If homogeneity was met, pairwise comparisons were conducted using Dunnett's T3 and LSD‐t tests; if not, Dunnett's T3 test was employed.^60^ The statistical significance for group disparities was established at a threshold of *p* < .05.

## 3 RESULTS

### 3.1 Comprehensive single‐cell RNA sequencing analysis and cell type identification in a mouse model of SSc

A SSc mouse model was established for the purpose of conducting scRNA‐seq analysis. The analysis revealed that a significant proportion of cells exhibited nFeature_RNA < 5000, nCount_RNA < 20000 and percent.mt < 20% (Figure S1A). The exclusion of low‐quality cells based on the 200 < nFeature_RNA < 5000 percent criteria.mt < 25%. It resulted in 22 959 genes and 14 836 cells in the expression matrix.

The sequencing depth analysis revealed that the correlation coefficient for filtered data showed a weak negative relationship (*r* = −0.12) between nCount_RNA and percent.mt, whereas a strong positive correlation (*r* = 0.93) was observed between nCount_RNA and nFeature_RNA (Figure S1B). This result indicates that the filtered cell data exhibit high quality and could proceed to the next analysis step.

Highly variable genes were identified through gene expression variance screening for further analysis on the filtered cells. Subsequently, downstream analysis focused on the top 2000 variable genes selected (Figure S1C). PCA performs linear dimensionality reduction on the data. The main gene expression heatmaps for PC_1 to PC_6 are presented in Figure S1D, accompanied by the cell distribution in PC_1 and PC_2, as depicted in Figure S1E. It was observed that there was no batch effect present in any of the samples.

**FIGURE 1:**
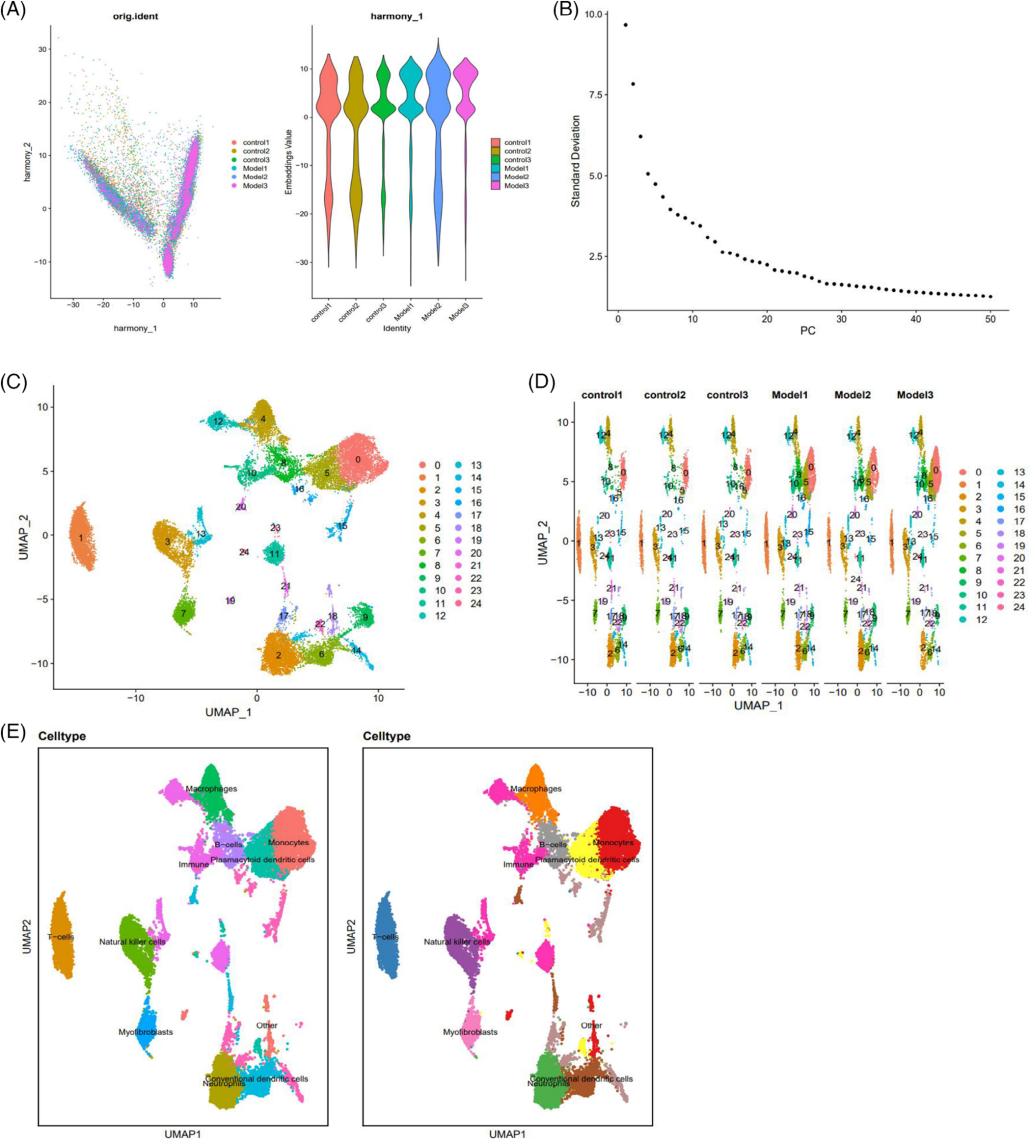
Cell clustering and annotation of scRNA‐seq data. Note: (A) Distribution of cells after batch correction in PC_1 and PC_2, each dot represents a cell. (B) Distribution of standard deviation of PCs, important PCs have larger standard deviation. (C) UMAP visualisation of clustering results, showing the aggregation and distribution of cells from different sources, with each colour representing a cluster. (D) UMAP visualisation of clustering results, showing the aggregation and distribution of cells from different sources, with each colour representing a cluster. (E) Visualisation of cell annotation results based on UMAP clustering, with each colour representing a cell cluster.

In order to improve the precision of cell clustering and diminish the influence of batch effects, the study deployed the Harmony software package for batch correction on the sample data (Figure S1F). The corrected results indicate that the batch effect in the samples has been successfully eliminated (Figure 1A). ElbowPlot was employed to rank the PCs based on their standard deviation. The results suggest that PCs PC_1 to PC_20 effectively capture the information of highly variable genes and hold analytical relevance (Figure 1B).

Furthermore, we applied the UMAP algorithm to reduce nonlinear dimensionality on the initial set of 20 PCs. We obtained 25 cell clusters through UMAP clustering (Figure 1C–D). Subsequently, we utilised the Bioconductor/R software package ‘SingleR’ to automatically annotate these 25 cell clusters, resulting in the identification of nine cell types (Figure 1E). Cluster 0, 18, 19 were classified as monocytes, cluster 1 as T‐cells, cluster 2 as neutrophils, cluster 3 as natural killer cells, cluster 4 as macrophages, clusters 5, 22, 23, 24 as pDCs, clusters 6, 20, 21 as conventional dendritic cells, cluster 7 as myofibroblasts and cluster 8 as B‐cells (Figure 1E).

Based on the information above, the control and model groups could be partitioned into 25 cellular clusters, comprising a total of nine cellular subtypes.

### 3.2 Elucidating the pivotal role of pDCs in SSc through scRNA‐seq and intercellular signalling analysis

**FIGURE 2:**
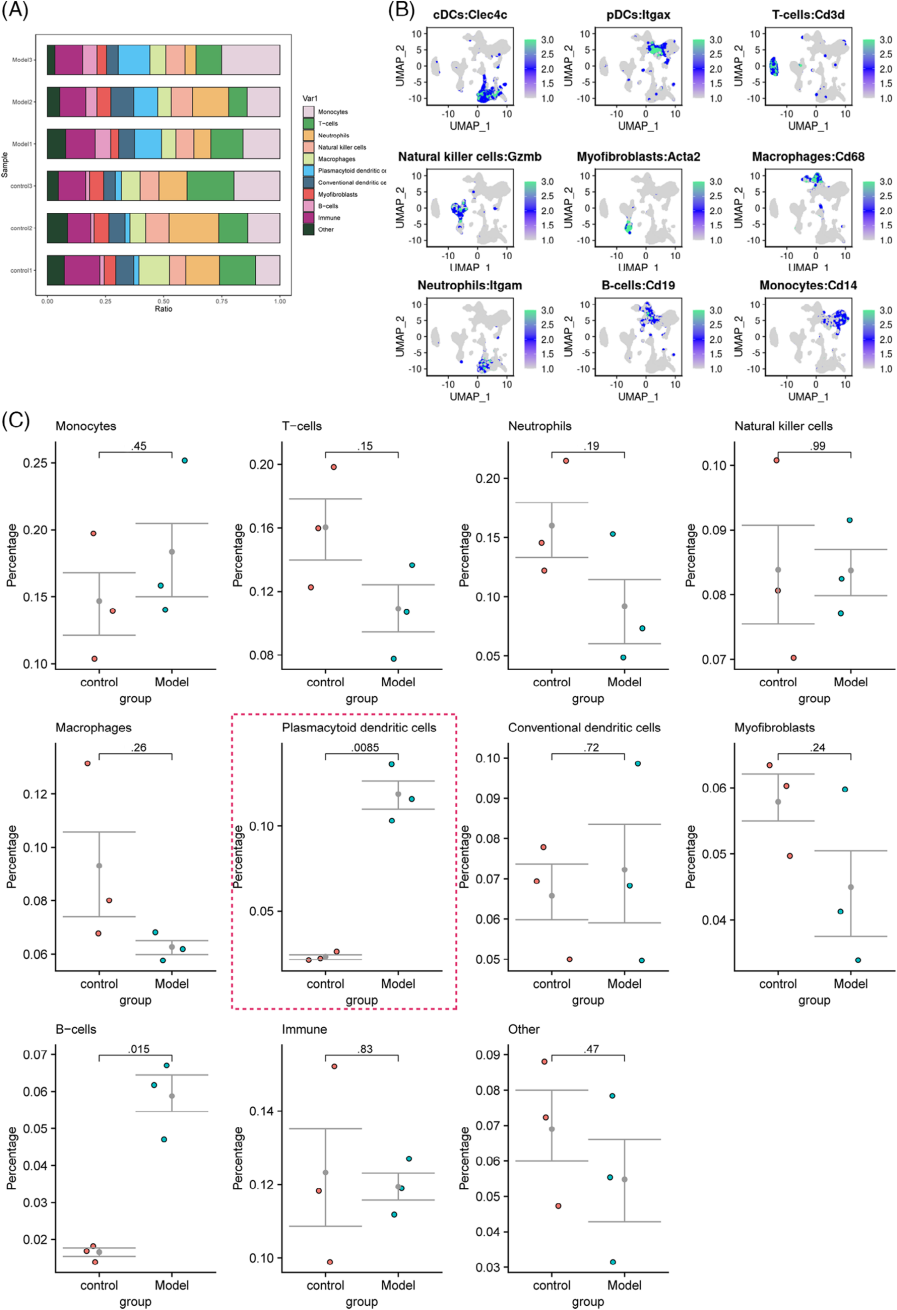
Analysis of the proportion of different cell types in normal and model lung tissue samples in SSc. (A) The proportion of different cell subtypes in each sample, with each subtype represented by a different colour. (B) Expression levels of the nine cell marker genes in each cell subtype, where darker blue indicates higher average expression levels. (C) *T*‐test analysis to assess the differential cell composition between the normal and model groups, with the purple box indicating the *T*‐test results for pDCs.

Multiple studies have demonstrated that the activation of pDCs triggers the generation of type I IFNs, thereby facilitating the development of a wide range of inflammatory and autoimmune ailments, such as SSc and systemic lupus erythematosus.^14^ We utilised Seurat to estimate the distribution of various cell types in each sample to explore the impact of pDCs in SSc (Figure 2A). Additionally, we visualised the UMAP expression maps of these nine cell marker genes. Cd14 serves as a marker gene for monocytes, Cd3d for T cells, Itgam (Cd11b) for neutrophils, Gzmb for natural killer cells, Cd68 for macrophages, Cd303 (Clec4c) for pDCs, Cd11c (Itgax) for conventional dendritic cells, Acta2 for myofibroblasts and Cd19 for B cells (Figure 2B).

We conducted *T*‐tests on lung tissue samples from both the control and experimental groups to assess the variations in cell type composition. The results indicate that pDCs have the lowest *p* value compared with other cell types, suggesting that the difference in content between normal and model group samples is greater in pDCs (Figure 2C). This finding suggests that pDCs may impact the development and progression of SSc.

To further investigate the functional differences contributing to cell number variations, we analysed the ‘CellChat’ package in the R language to explore intercellular signalling activities across different cell types. The results suggest an enhancement in the interaction between pDCs and macrophages, natural killer cells, neutrophils, T cells, B cells and monocytes in the model group (Figure S2A–D).

The results above indicate an increase in the content of pDCs in the experimental group samples In contrast to the control group samples. Additionally, there was an enhancement in the interaction between pDCs and macrophages, natural killer cells, T cells and B cells. It suggests that pDCs are crucial in developing autoimmune diseases. They potentially worsen the progression of these diseases by influencing the activity of other immune cells and immune responses.

### 3.3 Pseudotemporal dynamics and early activation of pDCs in autoimmune disease pathogenesis: insights from scRNA‐seq analysis

**FIGURE 3:**
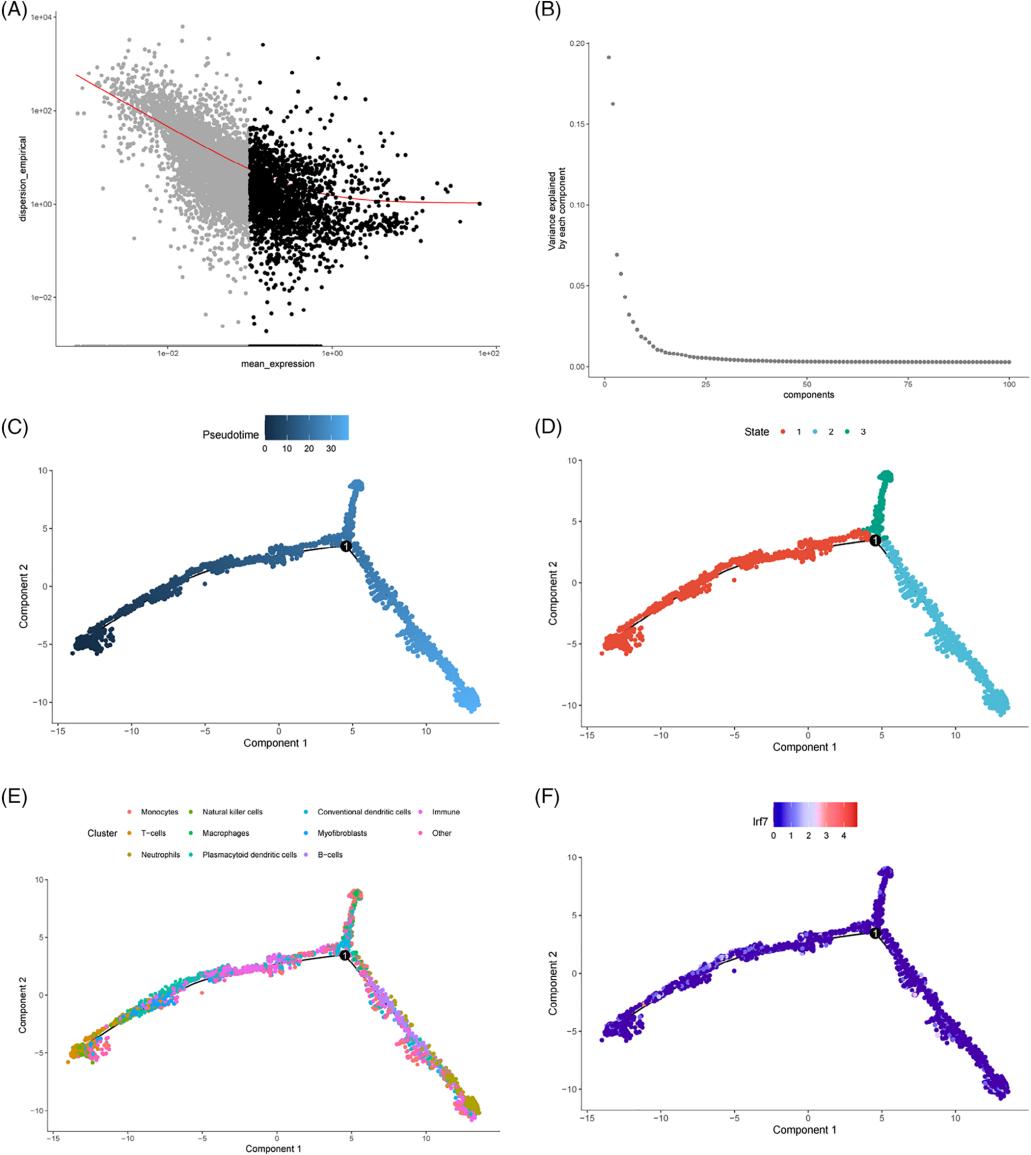
Pseudotime analysis of scRNA‐seq data. (A) Visualisation of sorted genes used for subsequent dimension reduction, each dot represents a gene. (B) PCA, with the *x*‐axis representing the component number and the *y*‐axis representing the proportion of variance explained. (C) Trajectory skeleton plot coloured by Pseudotime, with the darkness of the colour indicating the cell's rank based on pseudotime values. (D) Trajectory skeleton plot, with different colours representing different cell states of cell clusters. (E) Trajectory skeleton plot, with seurat cluster IDs mapped to the pseudotime order. (F) Expression changes of pDCs marker gene IRF7 in pseudotime.

We conducted a pseudo‐temporal analysis using the monocle2 R software package according to the scRNA‐seq data results. Figure 3A displays the visualisation of sorted genes. We utilised the DDRTree algorithm for reducing data dimensionality and sorted the cells relying on the expression trends of the sorting genes to construct trajectories, as depicted in Figure 3B. Pseudotime is a probability value calculated by Monocle 2 using cell gene expression information. It depicts the chronological sequence, with the right side representing the tree's root and the left side representing the branches (Figure 3C). According to the State display, the evolution of cells could be categorised into three stages, with an important branching point (Figure 3D). Our findings indicate that pDCs are primarily in the State1 phase, as depicted in Figure 3E. We further confirmed the position of the marker gene IRF7 at the ‘onset’ stage in pseudotime by analysing its expression trend in pDCs (Figure 3F). This further elucidates that during the pathogenesis of autoimmune diseases, pDCs may serve as one of the initial immune system cells to respond, activating and guiding other immune cells to initiate a defence response.

### 3.4 Unravelling the impact of pDCs in bleomycin‐induced SSc: a comprehensive in vivo analysis

**FIGURE 4:**
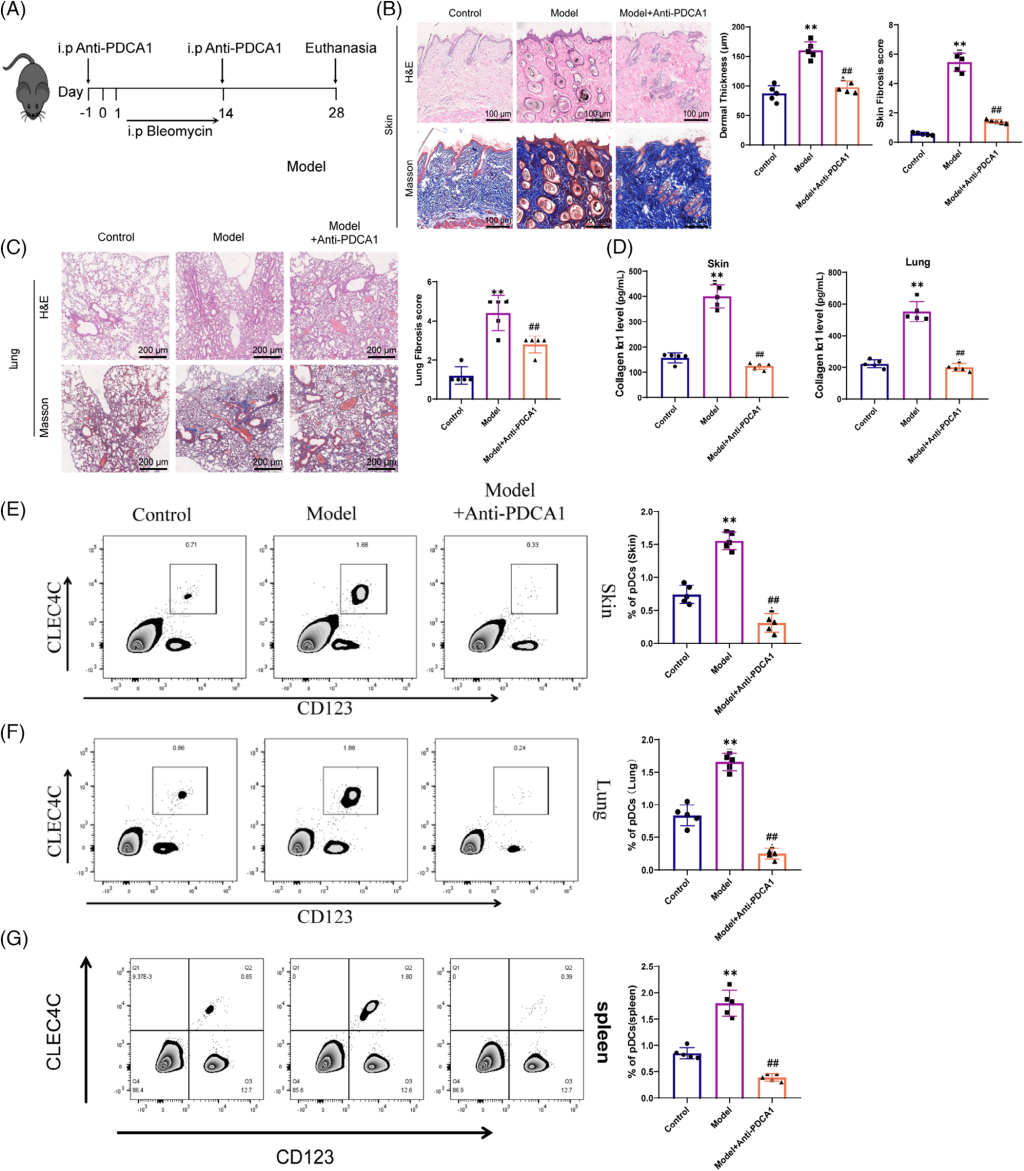
Effects of pDCs on animal models of SSc. (A) The method of bleomycin‐induced SSc animal model and PDCA‐1 antibody treatment. (B) H&E and Masson staining for pathological changes in skin tissue. (C) H&E and Masson staining for pathological changes in lung tissue. (D) ELISA to detect the expression levels of collagen Iα1 in skin and lung tissues. Tissues were lysed with cell lysis buffer, centrifuged and the supernatant was used for ELISA detection. (E) Flow cytometry for the expression levels of pDCs in skin tissues of each group. (F) Flow cytometry for the expression levels of pDCs in lung tissues of each group. (G) Flow cytometry for the expression levels of pDCs in spleen tissues of each group. Scale bar = 200 µm. Compared with the control group, **p < .01; compared with the model group, ##*p* < .01; animal experiments *n* = 5.

According to our scRNA‐seq analysis, we identified an increase in the content of pDCs in the lung tissue of mice in an SSc model (*p* < .01). To examine the in vivo mechanisms underlying the effects of pDCs in SSc disease, we employed a bleomycin‐induced SSc mouse model and administered 100 µg of PDCA‐1 antibody to them (Figure 4A).

Initially, the experiment employed H&E and Masson staining techniques to identify the pathological alterations in skin and lung tissues. The findings showed that, in relation to the control group, the model group displayed an augmentation in skin thickness, pronounced lung damage and substantial collagen deposition, noting the accomplishment in establishing the model. However, treatment with the PDCA‐1 antibody substantially relieved the skin and lung pathological manifestations in the model group while reducing collagen protein expression (Figure 4B,C). Furthermore, the ELISA test results revealed a significant increase in the expression of collagen Iα1 in the skin and lungs of the model group compared with the control group. Following treatment with PDCA‐1 antibody, there was a down‐regulation of collagen Iα1 expression in the lung and skin tissues (Figure 4D). Flow cytometry analysis revealed an increased proportion of CLEC4C+/CD123+ labelled pDCs in the skin, spleen and lungs of the model group contrary to the control group, indicating an accumulation of pDCs in this disease model. Following administration of anti‐PDCA‐1 antibody treatment, a notable decline in the proportion of pDCs expression was observed in comparison with the model group (Figure 4E–G).

In summary, our study findings highlight the critical role of pDCs in the development of bleomycin‐induced SSc disease, indicating their significance in the progression of skin and lung fibrosis. This underscores the pivotal involvement of pDCs in this disease model.

### 3.5 Deciphering the function of pDCs in SSc: a comprehensive gene expression analysis and identification of key regulatory molecules

**FIGURE 5:**
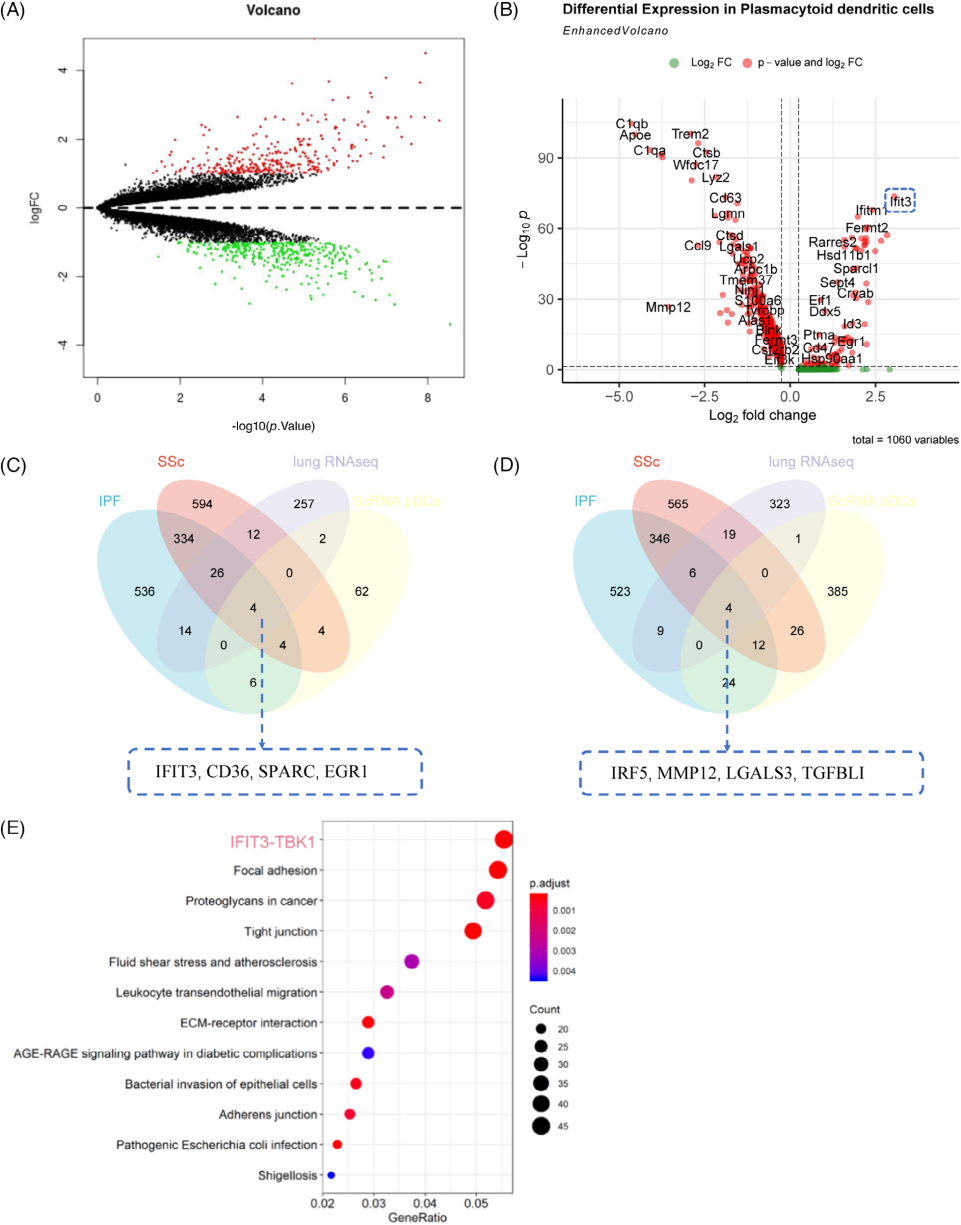
Analysis of key genes regulating SSc using combined scRNA‐seq and RNA‐seq. (A) Volcano plot of DEGs between normal and model groups analysed by RNA‐seq (*n* = 3), red represents up‐regulated genes, green represents down‐regulated genes, grey represents non‐significant genes. (B) Volcano plot of DEGs between pDCs in normal and model groups analysed by scRNA‐seq (*n* = 3), red dots to the left of the dashed line represent genes down‐regulated in the normal group, dots to the right of the dashed line represent genes up‐regulated in the model group. (C) Venn diagram showing the overlap of up‐regulated genes in RNA‐seq analysis, up‐regulated DEGs in pDCs, and SSc and IPF‐related genes downloaded from DisGeNET. (D) Venn diagram showing the overlap of down‐regulated genes in RNA‐seq analysis, down‐regulated DEGs in pDCs and SSc and IPF‐related genes downloaded from DisGeNET. (E) KEGG enrichment analysis.

pDCs, recognised as crucial immune cells, have been linked to the severity of lung disease in SSc patients.^35^ Consistent results were observed in our mouse experiments, indicating that the aggregation of pDCs exacerbates skin and lung fibrosis. High‐throughput transcriptomic sequencing revealed 678 DEGs between normal mouse lung tissue and model group mouse lung tissue, comprising 315 up‐regulated genes and 363 down‐regulated genes (Figure 5A).

In addition, we conducted a differential analysis of the gene expression profiles of pDCs in samples from both the control group and the model group. By applying a threshold of |avg_log2FC| > 0.5 and adj*p* < .05, we identified 82 genes with elevated expression in the model group and 458 genes with decreased expression. Notably, IFIT3 exhibited increased expression among these genes in the model group samples (Figure 5B).

Next, we compared the DEGs identified in pDCs through single‐cell sequencing and those obtained from RNA‐seq chips. Simultaneously, we obtained 940 genes associated with SSc and 936 genes associated with Interstitial Pulmonary Fibrosis (IPF) from the DisGeNET website. We then identified the common genes between the two. The results revealed a total of four up‐regulated intersectional genes, IFIT3, CD36, SPARC and EGR1 (Figure 5C), and four down‐regulated intersectional genes: IRF5, MMP12, LGALS3 and TGFBI (Figure 5D). Further KEGG pathway analysis revealed that IFIT3–TBK1 is the most significant signalling pathway (Figure 5E). Considering the differential expression from single‐cell sequencing analysis, RNA‐seq data and KEGG signalling pathway results, we determined that IFIT3 exhibits the most prominent differences, with significantly high expression in pDCs. Additionally, the IFIT3‐related signalling pathway, IFIT3–TBK1, emerged as the most significantly enriched KEGG pathway. Therefore, we selected IFIT3 as the focal point of this study.

### 3.6 Elucidating the regulatory mechanism of IFIT3–TBK1 axis in pDCs and its impact on SSc progression

IFIT3 exhibits antiviral activity and regulates the activation of TBK1.^22^ TBK1 is a kinase that can modulate multiple signalling pathways, and upon phosphorylation, it typically initiates the transcription of IFN‐I genes.^61^ During the immune response, pDCs secrete abundant IFN‐I upon extrinsic stimulation to mount an immune response.^62^ Therefore, we hypothesise that intervening in the IFIT3‐mediated phosphorylation of TBK1 (pTBK1) may inhibit the activation of pDCs, reduce IFN‐I production, and thereby alleviate the onset and progression of SSc.

To investigate the relationship between IFIT3 and TBK1, we conducted co‐immunoprecipitation experiments, revealing an interaction between IFIT3 and TBK1 (Figure S3A). Western blot analysis indicated an increase in the expression of IFIT3, TBK1 and pTBK1 in the skin and lungs of the model group in a juxtaposition with the control group (Figure S3B,C). This finding further emphasises the crucial role of IFIT3 and TBK1 in SSc.

**FIGURE 6:**
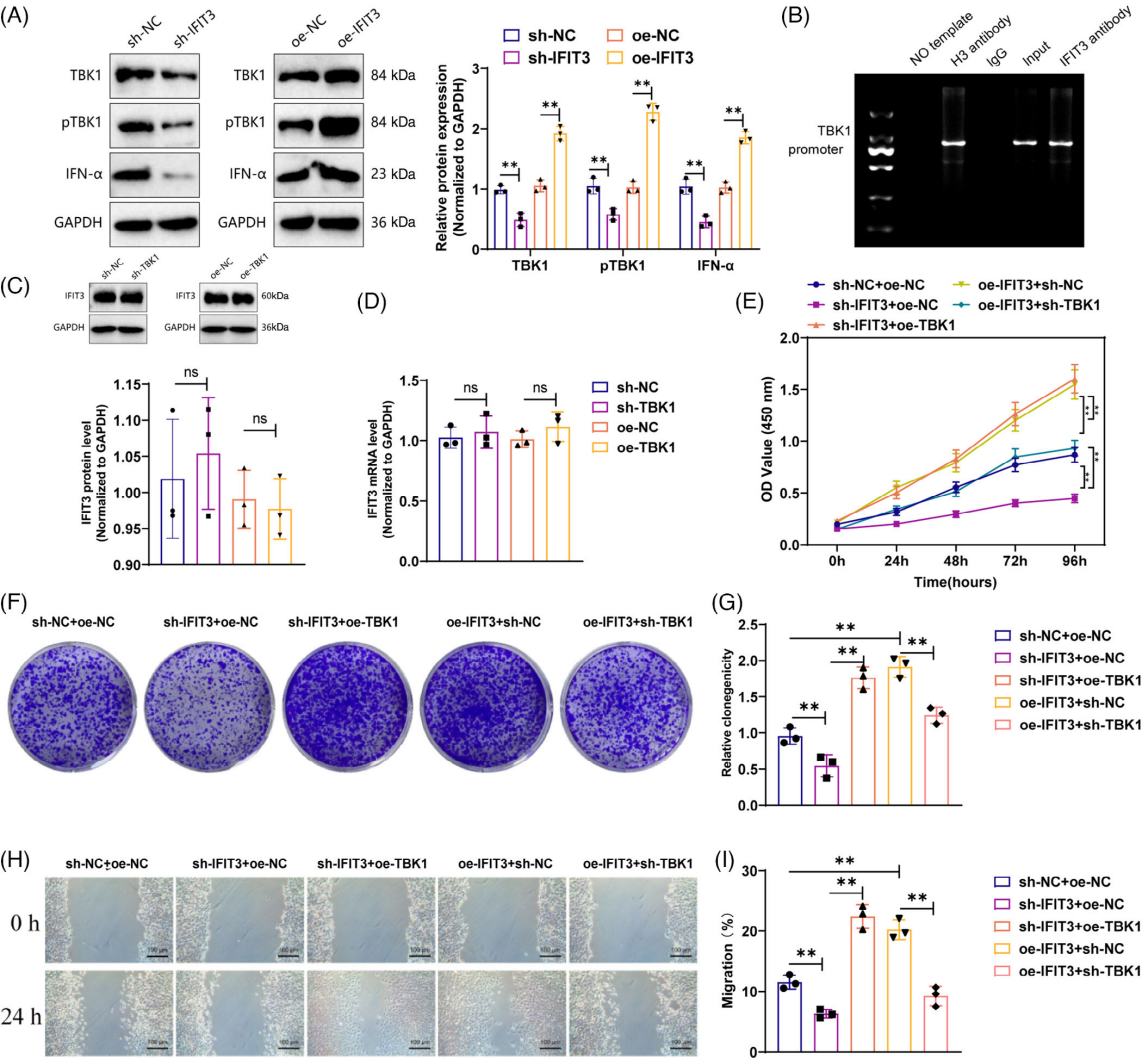
Activation of IFIT3 promotes pDCs activation by TBK1. (A) Silencing or overexpression of IFIT3 in pDCs was achieved in vitro, and Western blot was performed to detect the expression of TBK1, pTBK1 and IFN‐α. (B) ChIP was used to investigate the interaction between IFIT3 and TBK1. (C) Silencing or overexpression of TBK1 in pDCs was achieved in vitro, and Western blot was performed to detect the expression level of IFIT3 protein. (D) Silencing or overexpression of TBK1 in pDCs was achieved in vitro, and RT‐qPCR was performed to detect the expression level of IFIT3 mRNA. (E) CCK‐8 assay was performed to assess the impact of silencing or overexpression of IFIT3 and TBK1 on pDCs viability. (F and G) Colony formation assay was conducted to examine the effect of silencing or overexpression of IFIT3 and TBK1 on pDCs proliferation capacity. (H and I) Scratch assay was performed to investigate the effect of silencing or overexpression of IFIT3 and TBK1 on pDCs migration ability (Scale bar = 100 µm). **p < .01; ns *p* > .05, no statistically difference. Cell experiments were repeated three times.

To confirm the regulatory roles of IFIT3 and TBK1 on pDCs, we employed lentiviral constructs to establish pDCs cell models with silenced or overexpressed IFIT3. The qRT‐PCR and Western Blot results indicate successful establishment of an overexpression and knockdown system for IFIT3 (Figure S3D). The results indicated that TBK1, pTBK1 and IFN‐α expression were down‐regulated in the sh‐IFIT3 group compared with the sh‐NC group. Conversely, the proteins’ expression levels showed a rise in the oe‐IFIT3 group in contrast to the oe‐NC group (Figure 6A). These findings further support that IFIT3 could activate TBK1 to enhance the release of inflammatory factors in pDCs.

To further investigate the regulatory mechanism between IFIT3 and TBK1, we conducted ChIP experiments to verify. The experimental results demonstrate that IFIT3 can bind to the promoter of TBK1 (Figure 6B). Moreover, we generated a cellular model of pDCs with modulated expression levels of TBK1 (Figure S3E). Subsequently, we performed additional investigations using Western blot and RT‐qPCR experiments. The results demonstrate that neither silencing nor overexpression of TBK1 led to alterations in the expression levels of IFIT3 mRNA and protein (Figure 6C,D). Furthermore, this result suggests that IFIT3 controls the upstream factor involved in TBK1 activation, whereas TBK1 has no impact on the transcription of IFIT3.

Additionally, we examined the effect of the IFIT3/TBK1 pathway on the activity of pDCs. The viability of pDCs was found to be lower in the sh‐IFIT3+oe‐NC group than in the sh‐NC+oe‐NC group, as demonstrated by the CCK‐8 test outcomes. Conversely, the viability of pDCs was enhanced in the oe‐IFIT3+sh‐NC group. Moreover, the viability of pDCs was enhanced in the sh‐IFIT3+oe‐TBK1 group when comparing the sh‐IFIT3+oe‐NC group. Additionally, pDCs' viability decreased in the oe‐IFIT3+sh‐TBK1 group relative to the oe‐IFIT3+sh‐NC group (Figure 6E). The results of the colony formation assay and cell scratch assay revealed that, when juxtaposed with the sh‐NC+oe‐NC group, both the pDCs cell clone and migration ability declined in the sh‐IFIT3+oe‐NC group, while increased in the oe‐IFIT3+sh‐NC group. Moreover, the pDCs cell clone and migration ability improved in the sh‐IFIT3+oe‐TBK1 group in comparison with the sh‐IFIT3+oe‐NC group. Last, the pDCs cell clone and migration ability declined in the oe‐IFIT3+sh‐TBK1 group as opposed to the oe‐IFIT3+sh‐NC group (Figure 6F–I).

In conclusion, these experimental results emphasise the roles played by IFIT3 and TBK1 in pDCs. IFIT3 modulates the activation of TBK1, thereby impacting the biological functions of pDCs.

### 3.7 Deciphering the role of the IFIT3/TBK1 signalling pathway in pDC‐mediated fibroblast activation and fibrosis progression in SSc

SSc presents as an autoimmune condition impacting connective tissues, marked by abnormal immune system activity, which leads to excessive growth and fibrosis of connective tissues. This results in multi‐organ dysfunction.^3^ Various studies have demonstrated that when pDCs are over‐activated, they could stimulate fibroblasts, resulting in an enhanced release of cytokines and growth factors that exacerbate the fibrotic process.^63^ To deepen our understanding of the mechanisms by which pDCs induce fibrosis, we conducted experiments overexpressing IFIT3 and silencing TBK1. The experimental groups were as follows: oe‐NC+sh‐NC, oe‐IFIT3+sh‐NC, oe‐IFIT3+sh‐TBK1, where pDCs from these groups were respectively co‐cultured with fibroblasts.

**FIGURE 7:**
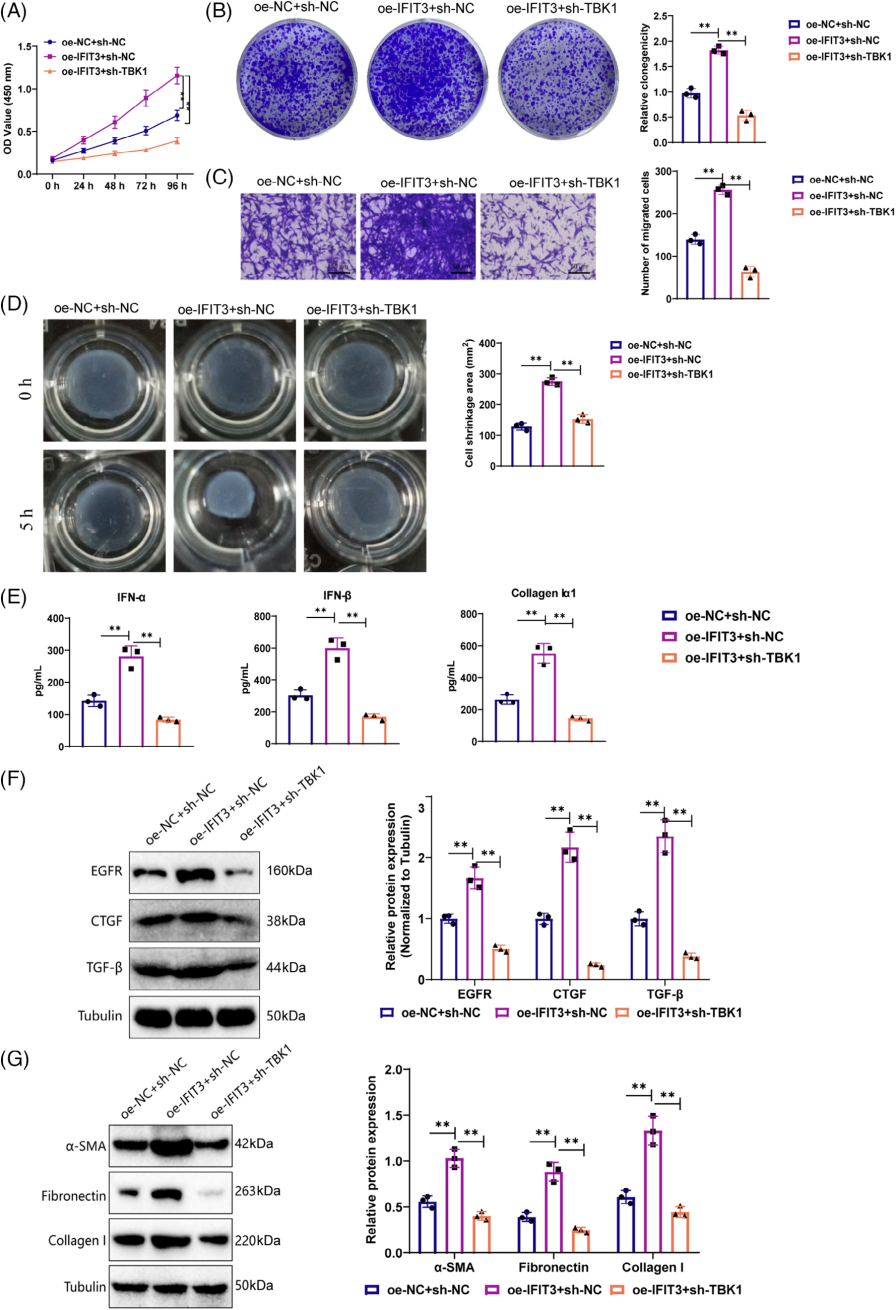
IFIT3/TBK1 signalling pathway regulates the impact of pDCs on fibroblast activity and factor expression. (A) Co‐culture of pDCs treated with oe‐NC+sh‐NC, oe‐IFIT3+sh‐NC, oe‐IFIT3+sh‐TBK1, with fibroblasts, followed by CCK‐8 assay to measure cell viability. (B) Colony formation assay to assess the proliferation capacity of pDCs treated with oe‐NC+sh‐NC, oe‐IFIT3+sh‐NC, oe‐IFIT3+sh‐TBK1, in co‐culture with fibroblasts. (C) Transwell assay to measure the migration ability of pDCs treated with oe‐NC+sh‐NC, oe‐IFIT3+sh‐NC, oe‐IFIT3+sh‐TBK1, in co‐culture with fibroblasts (Scale bar = 100 µm). (D) Contraction assay to evaluate the contraction ability of cells in different groups, including oe‐NC+sh‐NC, oe‐IFIT3+sh‐NC, oe‐IFIT3+sh‐TBK1, after 5 h of co‐culture with fibroblasts. (E) ELISA to detect the expression levels of collagen Iα1, IFN‐α and IFN‐β in the supernatant of the co‐culture system after 5 h. (F) Western blot to measure the expression levels of EGFR, CTGF and TGF‐β in the cells of different groups. (G) Western blot analysis to detect the expression levels of α‐SMA, fibronectin and collagen I in each group of cells. **p < .01; cell experiments were repeated three times.

The outcomes from the CCK‐8 experiments demonstrated that the oe‐IFIT3+sh‐NC group increased the viability of fibroblast cells in contrast with the oe‐NC+sh‐NC group. When juxtaposed with the oe‐IFIT3+sh‐NC group, the oe‐IFIT3+sh‐TBK1 group reduced the viability of fibroblasts (Figure 7A). The experimental results from colony formation and Transwell experiments demonstrated that the oe‐IFIT3+sh‐NC group enhanced fibroblast cells’ clonogenic and migratory abilities compared with the oe‐NC+sh‐NC group. Compared with the oe‐IFIT3+sh‐NC group, the oe‐IFIT3+sh‐TBK1 group demonstrated a reduction in fibroblasts’ cloning and migration ability (Figure 7B,C).

Cell contraction experiments demonstrated that the oe‐IFIT3+sh‐NC group progressed the contraction ability of fibroblasts contrasted with the oe‐NC+sh‐NC group. In contrast to the oe‐IFIT3+sh‐NC group, the oe‐IFIT3+sh‐TBK group demonstrated a suppression of fibroblast contraction (Figure 7D).

We utilised ELISA to measure the expression levels of collagen Iα1, IFN‐α and IFN‐β in the supernatant of the co‐culture system after a 5‐h duration. The results demonstrated that the expression levels of collagen Iα1, IFN‐α and IFN‐β were higher in the oe‐IFIT3+sh‐NC group as opposed to the oe‐NC+sh‐NC group. In the oe‐IFIT3+sh‐TBK1 group (Figure 7E), the expression levels of collagen Iα1, IFN‐α and IFN‐β were decreased when juxtaposed with the oe‐IFIT3+sh‐NC group.

Western blot analysis revealed that the oe‐IFIT3+sh‐NC group up‐regulated EGFR, CTGF, TGF‐β α‐SMA, fibronectin and collagen I expression levels, compared with the oe‐NC+sh‐NC group. In relation to the oe‐IFIT3+sh‐NC group, the expression levels of EGFR, CTGF, TGF‐β α‐SMA, fibronectin and collagen I were suppressed in the oe‐IFIT3+sh‐TBK1 group (Figure 7F,G).

As a summary, the analysis unveiled that the IFIT3/TBK1 signalling pathway influences the biological activity of fibroblasts in SSc. This pathway promotes the activation of pDCs, which results in the overexpression of fibrosis‐related factors and worsens the fibrotic process.

### 3.8 Elucidating the impact of IFIT3/TBK1 signalling pathway inhibition on SSc progression: in vivo insights from a CRISPR/Cas9‐induced knockout mouse model

**FIGURE 8:**
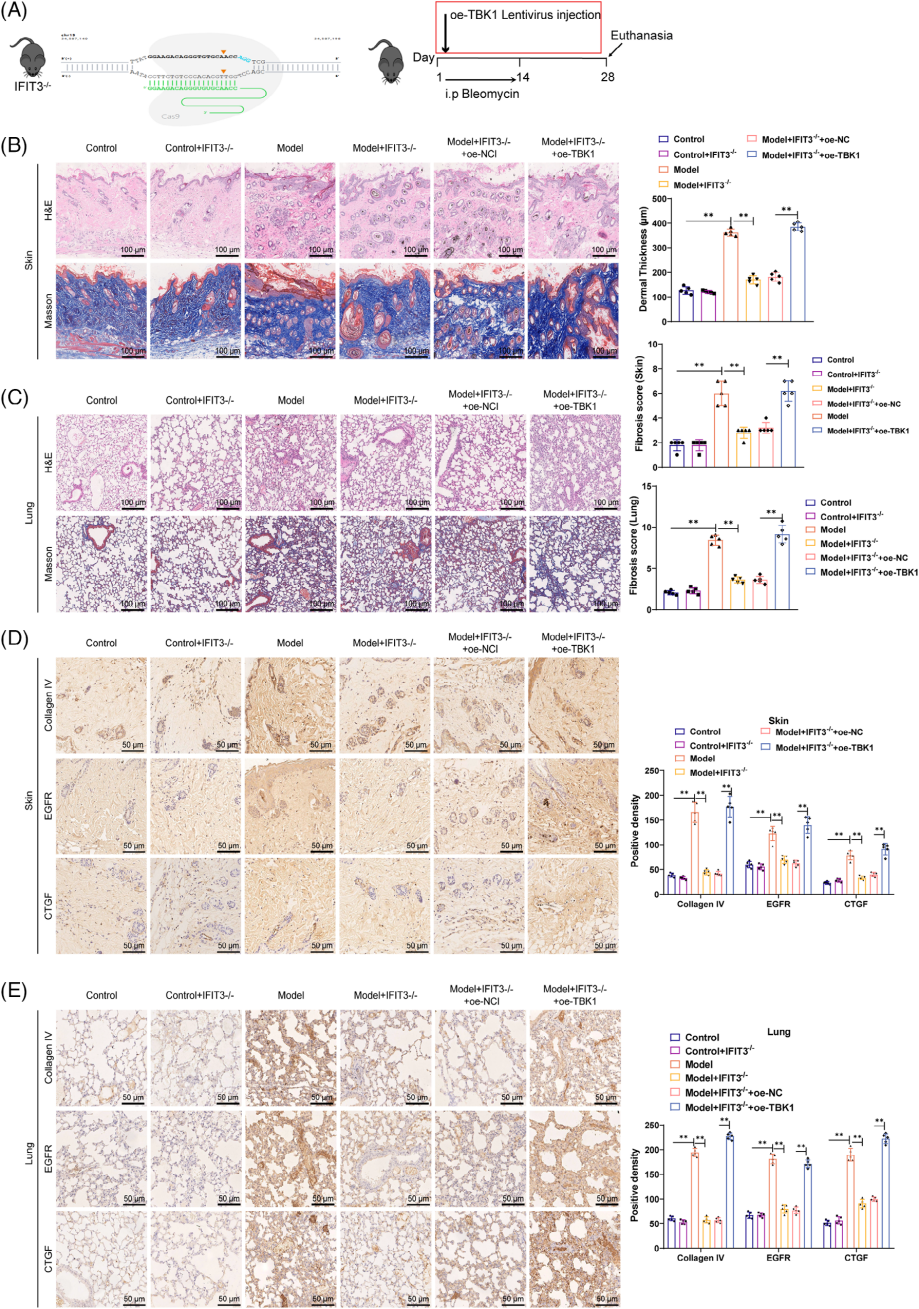
IFIT3 and TBK1 promote the progression of SSc. (A) CRISPR/Cas9 gene editing technique was used to knock out the IFIT3 gene in C57BL/6J mice, and a bleomycin‐induced SSc mouse model was established. (B) H&E staining and Masson staining were performed to evaluate the pathological status and collagen protein expression in the skin tissue. (C) H&E staining and Masson staining were performed to assess the pathological status and collagen protein expression in the lung tissue. (D) IHC staining was carried out to detect the expression of collagen IV, ECFR and CTGF in the skin tissue. (E) IHC staining was conducted to examine the expression of collagen IV, ECFR and CTGF in the lung tissue. Scale bar = 100 µm, **p < .05, animal experiment *n* = 5.

In order to investigate if similar effects could be observed in vivo, we utilised CRISPR/Cas9 editing technology to knockout the mouse IFIT3 gene (Figure S4A–C) and induced a SSc mouse model with bleomycin (Figure 8A).

The Western blot outcomes revealed a notable rise in the expression levels of IFIT3, TBK1 and pTBK1 in the model group, suggesting activation of the IFIT3/TBK1 signalling pathway. Conversely, in the model+IFIT3−/− group, the expression levels of these proteins were notably reduced, indicating that the absence of IFIT3 inhibited the activation of the IFIT3/TBK1 signalling pathway. However, upon administration of oe‐TBK1, the above phenomenon was reversed, with a marked up‐regulation in the expression levels of TBK1 and pTBK1 (Figure S4B,C).

The results of H&E staining and Masson staining suggested that, relative to the control group, the superficial layer of the skin thickened, and lung injury was severe in the model group, with evident collagen protein deposition. In contrast, the model+IFIT3−/− group exhibited improvement in the pathological condition of the skin and lung, accompanied by decreased collagen protein expression. However, the beneficial effect of IFIT3 knockout on skin and lung improvement in SSc‐associated PF mice was reversed by the model+IFIT3−/−+oe‐TBK1 group, in contrast to the model+IFIT3−/−+oe‐NC group (Figure 8B,C).

Additionally, the expression of collagen IV, EGFR and CTGF proteins in the skin and lungs was detected using immunohistochemistry (IHC). The results demonstrated an increase in the positive areas of collagen IV, EGFR and CTGF in the skin and lung of the model group as opposed to the control group. In contrast, the positive areas of these proteins were reduced in the model+IFIT3−/− group compared with the model group. Furthermore, the model+IFIT3−/−+oe‐TBK1 group exhibited an increase in the positive areas of collagen IV, EGFR and CTGF in the skin and lung compared with the model+IFIT3−/−+oe‐NC group (Figure 8D,E). These observations imply that inhibiting the IFIT3/TBK1 pathway activation could suppress the expression of fibrosis‐linked factors in the skin and lungs, thereby reducing the progression of fibrosis.

### 3.9 Modulating immune responses and inflammatory factors in SSc via inhibition of the IFIT3/TBK1 signalling pathway: in vivo evidence from a CRISPR/Cas9‐edited mouse model

**FIGURE 9:**
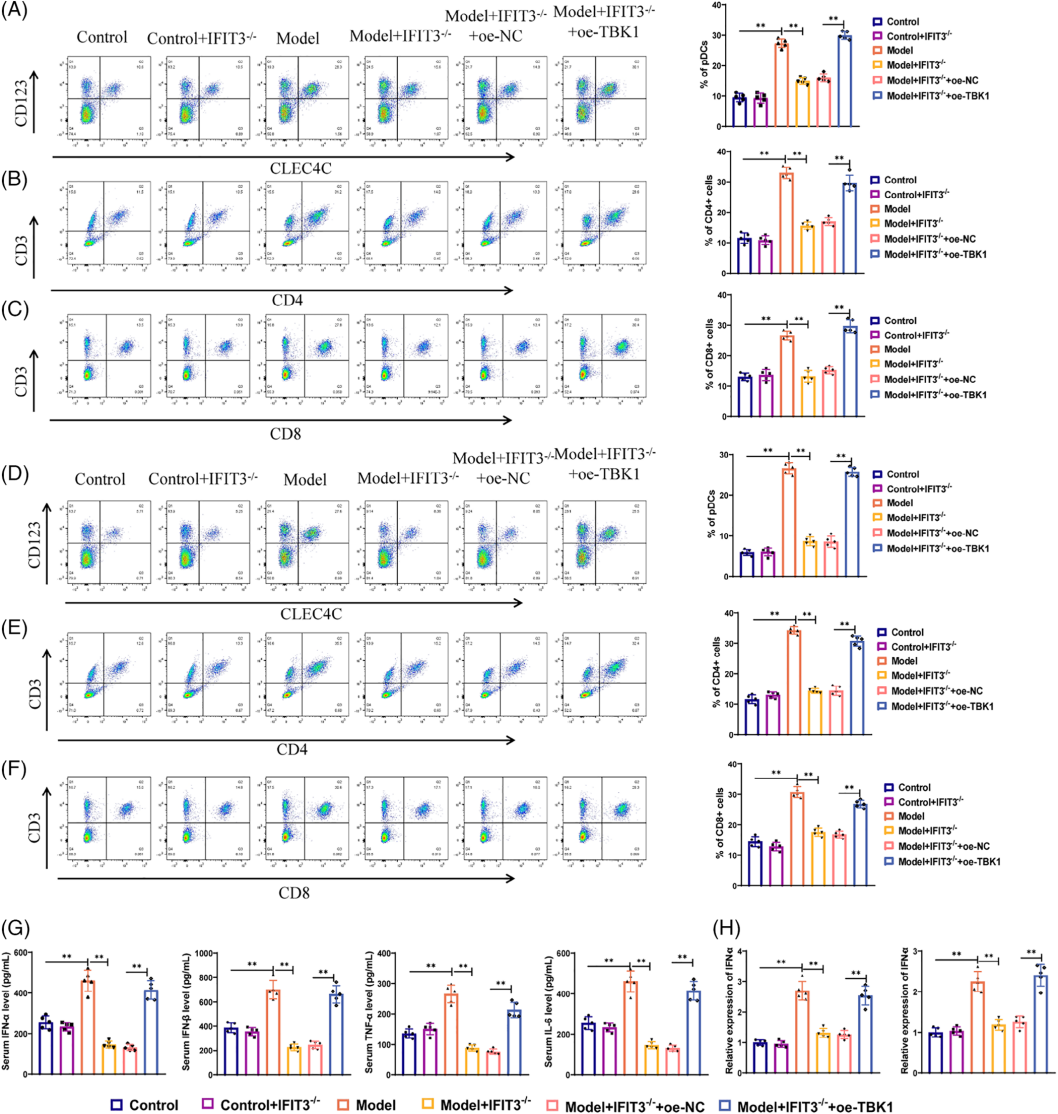
Intervention of IFIT3/TBK1 signalling pathway inhibits immune cell activation in SSc. (A–C) Flow cytometry was used to measure the expression levels of pDCs (A), CD4+T cells (B) and CD8+T cells (C) in the skin tissue. (D–F) Flow cytometry was performed to measure the expression levels of pDCs (D), CD4+T cells (E) and CD8+T cells (F) in the lung tissue. (G) ELISA was employed to detect the expression levels of the inflammatory factors IFN‐α, IFN‐β, TNF‐α and IL‐6 in peripheral blood. (H) Expression of IFN‐α in the skin and lungs. **p < .05; ns *p* > .05, no statistically difference, animal experiment *n* = 5.

During the immune response, pDCs recognise viruses or self‐nucleic acids through Toll‐like receptor 7 (TLR7) and Toll‐like receptor 9 (TLR9), which leads to the production of a variety of cytokines, including IFN‐α, IL‐6, CXCL8 and IL‐12. Moreover, through the action of MHC II or IL‐12, pDCs aid in the presentation of antigens to CD4+ T and CD8+ T cells, thereby triggering an immune response.^62^ Research in the clinical field has also proven that expression of cellular subsets, such as pDCs, CD4+ T cells, CD8+ T cells, and their associated factors in the skin and lung tissues of SSc patients, could contribute to the inflammation and fibrosis processes in SSc.^64^ In order to further investigate the mechanism underlying immune cell regulation in the IFIT3/TBK1 signalling pathway, we conducted experimental analysis employing flow cytometry. The results from the detection illustrated that the expression ratios of CD123+/CLEC4C+ pDCs, CD3+/CD4+ T cells and CD3+/CD8+ T cells in the skin and lung tissues of the model group were elevated in relation to the control group. The expression ratios of CD123+/CLEC4C+ pDCs, CD3+/CD4+ T cells and CD3+/CD8+ T cells were lower in the model+IFIT3−/− group in comparison with the model group. Contrasted with the model+IFIT3−/−+oe‐NC group, the expression levels of the cells above in the skin and lung tissues increased in the model+IFIT3−/−+oe‐TBK1 group (Figure 9A–F).

Additionally, we employed ELISA to examine the levels of inflammatory factors in the serum. The test results indicate an increase in the expression of IFN‐α, IFN‐β, TNF‐α and IL‐6 in the model group relative to the control group. Compared with the model group, the expression levels of IFN‐α, IFN‐β, TNF‐α and IL‐6 were reduced in the model+IFIT3−/− group. Expression of the aforementioned inflammatory factors was up‐regulated in the model+IFIT3−/−+oe‐TBK1 group as opposed to the model+IFIT3−/−+oe‐NC group (Figure 9G). We also performed qPCR to analyse the expression of IFN‐α in the lung and skin, with the results consistent with the serum findings (Figure 9H).

In conclusion, the results suggest that inhibiting the IFIT3/TBK1 signalling pathway could effectively suppress the triggering of pDCs and the liberation of inflammatory factors, thus alleviating the progression of SSc.

## 4 DISCUSSION

This study uncovers a substantial role of the IFIT3/TBK1 signalling pathway in SSc, specifically in activating pDCs. Consistent with previous studies, the analysis of our data leads us to the conclusion that the involvement of pDCs is pivotal in the advancement of SSc.^13^, ^14^ IFIT3, as an upstream regulator of TBK1 activation, could influence the development of SSc by affecting the activation of pDCs.^61^ In this study, we have provided additional insights into the specific regulatory mechanism of IFIT3 on TBK1. This viewpoint is substantiated by compelling evidence obtained from various cellular biology experiments. This paper offers a novel perspective compared with prior research on the IFIT3/TBK1 signalling pathway, suggesting that intervening in this pathway could effectively suppress the activation of pDCs. Previous research has primarily concentrated on the essential role played by the IFIT3/TBK1 signalling pathway in the context of antiviral responses.^17^, ^65^, ^66^ This study presents and validates the proposal that IFIT3 regulates the activation of TBK1, consequently impacting the activity of pDCs and influencing the progression of SSc.

The literature has reported the involvement of IFIT3 in various pathological processes, including certain inflammatory responses and tumour development.^67^, ^68^, ^69^ This study offers a distinctive viewpoint on the function and regulatory mechanisms of IFIT3 in SSc, specifically its involvement in the fibrotic process. Furthermore, it reveals a diverse IFIT3 landscape that differs from its role in other diseases. Throughout the progression of SSc, pDCs are not solely restricted to interacting with the IFIT3/TBK1 signalling pathway. They might also play a role in regulating various other signalling pathways.^61^ While this study primarily investigates the IFIT3/TBK1 signalling pathway, further research should explore the potential role of pDCs in various other aspects, such as their interactions with different signalling pathways and molecules and their involvement in other pathological processes. This avenue of investigation holds promise for future investigations.

The knockout mouse model of the IFIT3 gene, constructed using CRISPR/Cas9 gene editing technology, is a valuable tool for investigating the in vivo function of IFIT3.^70^ In the future, optimising CRISPR technology and its application in constructing more complex disease models will further enhance our understanding of intricate diseases like SSc. Our study demonstrated that intervention in the IFIT3/TBK1 signalling pathway effectively reduces fibrosis in the mouse model of SSc, suggesting a promising direction for future drug development to target this pathway. Nevertheless, the process of translating these laboratory findings into clinical applications, while ensuring safety and efficacy in human patients, necessitates further basic and clinical research.

**FIGURE 10:**
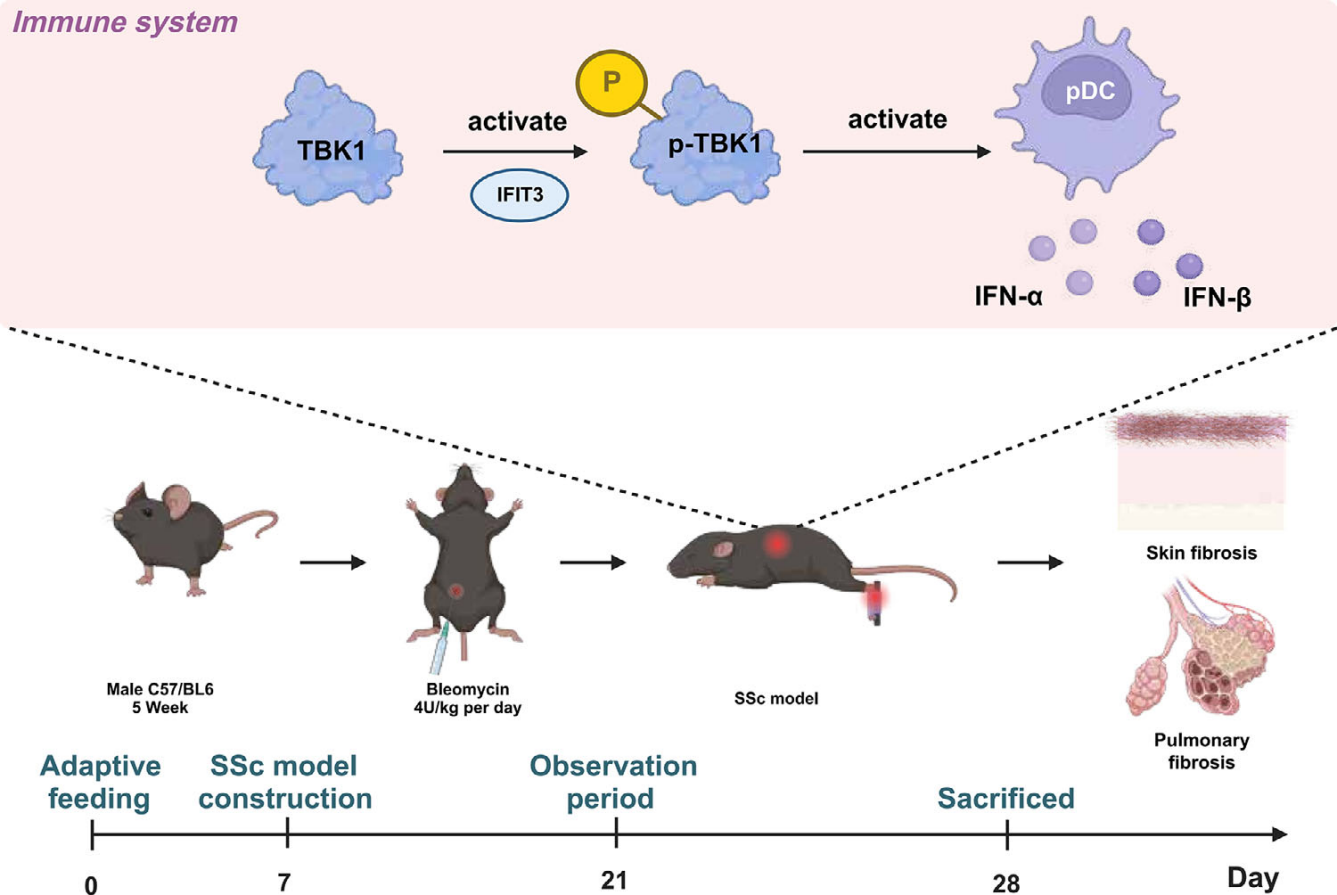
Schematic diagram illustrating the molecular mechanism of IFIT3‐mediated activation of pDCs through TBK1 phosphorylation to promote fibrosis.

This study investigates the potential mechanisms through which IFIT3/TBK1 regulates pDCs in SSc, utilising scRNA‐seq, molecular biology techniques and animal experiments. We annotated nine cell types using single‐cell transcriptomic sequencing and identified IFIT3 as a highly expressed gene, specifically in pDCs. Alleviation of the severity of SSc could be achieved by intervening in the IFIT3/TBK1 signalling pathway to inhibit the stimulation of pDCs and reduce the liberation of inflammatory factors, as illustrated in Figure 10. Our research has uncovered the crucial role and regulatory mechanism of the IFIT3/TBK1 signalling pathway in pDC activation and SSc. These findings suggest potential research directions and treatment strategies for future SSc therapies.

This study explores the IFIT3/TBK1 signalling pathway's regulatory mechanism in activating pDCs in SSc. This groundbreaking discovery has the potential to uncover new research avenues and offer novel therapeutic targets for SSc treatment. This study employs scRNA‐seq and other advanced techniques to explore the potential function of the IFIT3 gene in SSc. Understanding the pathological and physiological mechanisms of SSc is improved by recognising the crucial role of IFIT3 in activating TBK1 and pDCs and its influence on fibroblast contraction, proliferation, migration and differentiation abilities. This study found an increase in type I IFN in SSc mice. It has been documented in prior studies that the elevation of IFN may correlate with fibrosis formation. Liu et al.^71^ documented that in vitro, fibroblasts respond to macrophage‐derived DNA and the activation of the type I IFN response occurs via the STING/TBK1/IRF3 signalling pathway. Moreover, under conditions of chronic stimulation, the fibrogenic consequences of cytoplasmic DNA and type I IFN seem to be correlated with fibroblast activation, whereas sudden stimulation of fibroblasts by DNA and type I IFN could induce an anti‐fibrotic state.^71^ Furthermore, it was discovered that intervening in the IFIT3/TBK1 signalling pathway could reduce fibrosis in mice's skin and lungs with SSc. This finding offers promising prospects for future clinical treatment of SSc. Clinically, IFIT3 serves as a potential therapeutic target and could hold a pivotal position in the future therapeutic approach to SSc. Numerous existing SSc treatment methods aim to alleviate symptoms rather than address the underlying causes. Consequently, the discovery and intervention research of the IFIT3/TBK1 signalling pathway presents a promising approach to directly target the aetiology and potentially impede or even halt the progression of SSc.^72^

This study provides a localised perspective on SSc, focusing on the involvement of the IFIT3/TBK1 signalling pathway in pDC activation and fibrosis processes. Integrating these localised cellular and molecular findings into a broader framework, such as combining them with various factors like the immune environment, genetic predisposition and environmental influences, will further enhance our understanding of SSc. Overall, this research not only enriches our comprehension of SSc, offering potential therapeutic targets, but also expands our knowledge of the significance of the IFIT3/TBK1 signalling pathway in immune regulation and pathological processes. Further exploration on the conditional knockout or overexpression of IFIT3 or TBK1 in mouse pDCs warrants in‐depth investigation. Subsequent in‐depth studies and clinical validation will drive the translation of these fundamental research findings into clinical practice.

Although this study had some promising findings, it also had some limitations. First, while we did observe the impact of IFIT3 knockout on the progression of SSc in the mouse model, it is crucial to acknowledge that the mouse model does not completely mirror human disease. As a result, these findings should be validated in supplementary experimental models and human samples. Furthermore, while the primary focus of this study is on the involvement of the IFIT3/TBK1 signalling pathway in activating pDCs, it is crucial not to overlook the role of other immune and non‐immune cells in SSc, along with their respective signalling pathways. Therefore, these aspects should be further explored. Although CRISPR/Cas9 gene editing has provided a convenient method for gene knockout, the potential off‐target effects and long‐term safety require further investigation. This study confirmed that IFIT3−/− mice exhibited attenuated skin and lung fibrosis induced by bleomycin. However, additional investigations are needed to determine whether overexpression of IFIT3 can directly induce skin and lung fibrosis in mice. Future research should delve into the detailed molecular mechanisms of the IFIT3/TBK1 signalling pathway and explore other potential synergistic molecules and pathways. Furthermore, expanding the study to include more animal models and human samples, particularly across different stages and subtypes of SSc, focusing on cellular studies of SSc patients, is crucial to validate our findings and delve deeper into their universality and significance in the pathogenesis of SSc.

Drug development based on the IFIT3/TBK1 signalling pathway may be a promising direction in terms of clinical applications. There is a need to develop specific and low‐side‐effect small molecule compounds or biopharmaceuticals to target the IFIT3/TBK1 signalling pathway. Subsequent pharmacological and toxicological evaluations should be performed using large animal models, followed by preliminary clinical trials. These steps will offer new prospects for the clinical treatment of SSc. Furthermore, the significance of this signalling pathway in other autoimmune diseases merits further investigation, offering the potential for its wide‐ranging clinical application.

## AUTHOR CONTRIBUTIONS

Xiangyang Huang and Xia Rong contributed equally as co‐first authors to this study. Xiangyang Huang conceived and designed the research project. Xiangyang Huang, Xia Rong, Yiheng Zhao, Dan Feng and Jun Wang conducted the experiments and collected the data. Xiangyang Huang and Xia Rong performed the data analysis. Xiangyang Huang and Xia Rong contributed to the manuscript writing. All authors critically reviewed and approved the final version of the manuscript.

## CONFLICT OF INTEREST STATEMENT

The authors declare no conflict of interest.

## ETHICS STATEMENT

All animal experiments conducted in this study were ethically reviewed and approved by the Animal Ethics Committee of West China School of Public Health and West China Fourth Hospital, Sichuan University. These experiments were carried out in strict accordance with the established ethical guidelines and principles for the care and use of laboratory animals. All efforts were made to ensure minimal suffering and the highest standards of animal welfare throughout the research process.

## CONSENT TO PARTICIPATE

Not applicable.

## CONSENT FOR PUBLICATION

Not applicable.

## Supporting information

Figure S1. Data quality control and PCA dimensionality reduction of scRNA‐seq.
Note: (A) Violin plots showing the number of genes (nFeature_RNA), mRNA molecules (nCount_RNA), and percentage of mitochondrial genes (percent.mt) in each cell of the scRNA‐seq data. (B) Scatter plots depicting the correlation between filtered data nCount_RNA and percent.mt, as well as nCount_RNA and nFeature_RNA. (C) Differential expression analysis to identify highly variable genes, with red indicating the top 2000 highly variable genes and black representing genes with low variability. The top 10 gene names from the highly variable gene set are labelled. (D) Heatmap displaying the expression of the top 20 genes most correlated with PC_1 ‐ PC_6 in the PCA, with yellow indicating upregulation and purple indicating downregulation. (E) Distribution of cells before batch correction in PC_1 and PC_2, with each point representing a cell. (F) Batch correction process diagram using Harmony, where the x‐axis represents the number of iterations.

Figure S2. Analysis of cell‐cell interactions in scRNA‐seq.
Note: (A) Circular plot showing cell‐cell interactions in the normal group, with line thickness representing interaction strength. (B) Cell‐cell interactions of pDCs with other cells in the normal group. (C) Circular plot depicting cell‐cell interactions in the model group, with line thickness representing interaction strength. (D) Cell‐cell interactions of pDCs with other cells in the model group.

Figure S3. Involvement of IFIT3 and TBK1 in the development of SSc.
Note: (A) Co‐immunoprecipitation experiment of IFIT3 and TBK1; (B) Expression levels of IFIT3, TBK1, and pTBK1 in skin tissue detected by Western blot in the Bleomycin‐induced SSc mouse model. (C) Expression levels of IFIT3, TBK1, and pTBK1 in lung tissue were detected by Western blot in the Bleomycin‐induced SSc mouse model. (D) Transfection efficiency of silencing or overexpressing IFIT3 detected by RT‐qPCR and Western Blot. (E) Transfection efficiency of silencing or overexpressing TBK1 detected by RT‐qPCR. *P < 0.01, P < 0.05, animal experiments n = 5, cell experiments repeated 3 times.

Figure S4. Inhibition of TBK1 activation and release of inflammatory factors by IFIT3.
Note: (A) Mouse gene typing data. M represents Marker, 1 and 4 represent IFIT3−/− mice, 2 and 3 represent wild‐type mice; (B) Expression levels of IFIT3, TBK1, and pTBK1 in skin tissue detected by Western blot. (C) Western blot detected expression levels of IFIT3, TBK1, and pTBK1 in lung tissue. **P < 0.05, animal experiments n = 5.

## Data Availability

The data underlying this article will be shared on reasonable request to the corresponding author.

## References

[1] Bobeica C , Niculet E , Musat CL , et al. Paraclinical aspects in systemic sclerosis. Int J Gen Med. 2022;15:4391‐4398. doi:10.2147/IJGM.S355662 35502184 PMC9056056

[2] Jerjen R , Nikpour M , Krieg T , Denton CP , Saracino AM . Systemic sclerosis in adults. Part I: clinical features and pathogenesis. J Am Acad Dermatol. 2022;87(5):937‐954. doi:10.1016/j.jaad.2021.10.065 35131402

[3] Denton CP , Khanna D . Systemic sclerosis. Lancet. 2017;390(10103):1685‐1699. doi:10.1016/S0140-6736(17)30933-9 28413064

[4] Stochmal A , Czuwara J , Trojanowska M , Rudnicka L . Antinuclear antibodies in systemic sclerosis: an update. Clin Rev Allergy Immunol. 2020;58(1):40‐51. doi:10.1007/s12016-018-8718-8 30607749

[5] Sobolewski P , Maślińska M , Wieczorek M , et al. Systemic sclerosis—multidisciplinary disease: clinical features and treatment. Reumatologia. 2019;57(4):221‐233. doi:10.5114/reum.2019.87619 31548749 PMC6753596

[6] Tuhy T , Hassoun PM . Clinical features of pulmonary arterial hypertension associated with systemic sclerosis. Front Med (Lausanne). 2023;10:1264906. doi:10.3389/fmed.2023.1264906 37828949 PMC10565655

[7] Kopp JB , Anders HJ , Susztak K , et al. Podocytopathies. Nat Rev Dis Primers. 2020;6(1):68. doi:10.1038/s41572-020-0196-7 32792490 PMC8162925

[8] Baharom F , Ramirez‐Valdez RA , Khalilnezhad A , et al. Systemic vaccination induces CD8^+^ T cells and remodels the tumor microenvironment. Cell. 2022;185(23):4317‐4332. doi:10.1016/j.cell.2022.10.006 e15 36302380 PMC9669246

[9] Fang D , Chen B , Lescoat A , Khanna D , Mu R . Immune cell dysregulation as a mediator of fibrosis in systemic sclerosis. Nat Rev Rheumatol. 2022;18(12):683‐693. doi:10.1038/s41584-022-00864-7 36352098

[10] Farge D , Loisel S , Lansiaux P , Tarte K . Mesenchymal stromal cells for systemic sclerosis treatment. Autoimmun Rev. 2021;20(3):102755. doi:10.1016/j.autrev.2021.102755 33476823

[11] Worrell JC , O'Reilly S . Bi‐directional communication: conversations between fibroblasts and immune cells in systemic sclerosis. J Autoimmun. 2020;113:102526. doi:10.1016/j.jaut.2020.102526 32713676

[12] Lee‐Sundlov MM , Burns RT , Kim TO , et al. Immune cells surveil aberrantly sialylated O‐glycans on megakaryocytes to regulate platelet count. Blood. 2021;138(23):2408‐2424. doi:10.1182/blood.2020008238 34324649 PMC8662070

[13] Silva IS , Ferreira BH , Almeida CR . Molecular mechanisms behind the role of plasmacytoid dendritic cells in systemic sclerosis. Biology (Basel). 2023;12(2):285. doi:10.3390/biology12020285 36829561 PMC9953616

[14] Chaudhary V , Ah Kioon MD , Hwang SM , et al. Chronic activation of pDCs in autoimmunity is linked to dysregulated ER stress and metabolic responses. J Exp Med. 2022;219(11):e20221085. doi:10.1084/jem.20221085 36053251 PMC9441715

[15] Onuora S . Targeting pDCs in SSc. Nat Rev Rheumatol. 2021;17(4):189. doi:10.1038/s41584-021-00598-y 33686280

[16] Huang Y , Li X , Sun X , et al. Anatomical transcriptome atlas of the male mouse reproductive system during aging. Front Cell Dev Biol. 2022;9:782824. doi:10.3389/fcell.2021.782824 35211476 PMC8861499

[17] Zhang W , Li Y , Xin S , et al. The emerging roles of IFIT3 in antiviral innate immunity and cellular biology. J Med Virol. 2023;95(1):e28259. doi:10.1002/jmv.28259 36305096

[18] Franco JH , Chattopadhyay S , Pan ZK . How different pathologies are affected by IFIT expression. Viruses. 2023;15(2):342. doi:10.3390/v15020342 36851555 PMC9963598

[19] Shen M , Duan C , Xie C , et al. Identification of key interferon‐stimulated genes for indicating the condition of patients with systemic lupus erythematosus. Front Immunol. 2022;13:962393. doi:10.3389/fimmu.2022.962393 35967341 PMC9365928

[20] Luo H , Zhou X . Bioinformatics analysis of potential common pathogenic mechanisms for COVID‐19 infection and primary Sjogren's syndrome. Front Immunol. 2022;13:938837. doi:10.3389/fimmu.2022.938837 35958619 PMC9360424

[21] Huijser E , Bodewes ILA , Lourens MS , et al. Hyperresponsive cytosolic DNA‐sensing pathway in monocytes from primary Sjögren's syndrome. Rheumatology (Oxford). 2022;61(8):3491‐3496. doi:10.1093/rheumatology/keac016 35022662 PMC9348764

[22] Liu XY , Chen W , Wei B , Shan YF , Wang C . IFN‐induced TPR protein IFIT3 potentiates antiviral signaling by bridging MAVS and TBK1. J Immunol. 2011;187(5):2559‐2568. doi:10.4049/jimmunol.1100963 21813773

[23] Meng W , Gao SJ . Targeting XPO1 enhances innate immune response and inhibits KSHV lytic replication during primary infection by nuclear stabilization of the p62 autophagy adaptor protein. Cell Death Dis. 2021;12(1):29. doi:10.1038/s41419-020-03303-1 33414399 PMC7790339

[24] Chikhalya A , Dittmann M , Zheng Y , Sohn SY , Rice CM , Hearing P . Human IFIT3 protein induces interferon signaling and inhibits adenovirus immediate early gene expression. mBio. 2021;12(6):e0282921. doi:10.1128/mBio.02829-21 34724821 PMC8561380

[25] Jiang M , Jia K , Wang L , et al. Alterations of DNA damage response pathway: biomarker and therapeutic strategy for cancer immunotherapy. Acta Pharm Sin B. 2021;11(10):2983‐2994. doi:10.1016/j.apsb.2021.01.003 34729299 PMC8546664

[26] Oduro PK , Zheng X , Wei J , et al. The cGAS‐STING signaling in cardiovascular and metabolic diseases: future novel target option for pharmacotherapy. Acta Pharm Sin B. 2022;12(1):50‐75. doi:10.1016/j.apsb.2021.05.011 35127372 PMC8799861

[27] Chen R , Du J , Zhu H , Ling Q . The role of cGAS‐STING signalling in liver diseases. JHEP Rep. 2021;3(5):100324. doi:10.1016/j.jhepr.2021.100324 34381984 PMC8340306

[28] Sun Y , Revach OY , Anderson S , et al. Targeting TBK1 to overcome resistance to cancer immunotherapy. Nature. 2023;615(7950):158‐167. doi:10.1038/s41586-023-05704-6 36634707 PMC10171827

[29] Shao W , Todd TW , Wu Y , et al. Two FTD‐ALS genes converge on the endosomal pathway to induce TDP‐43 pathology and degeneration. Science. 2022;378(6615):94‐99. doi:10.1126/science.abq7860 36201573 PMC9942492

[30] Liu Y , Xu P , Rivara S , et al. Clathrin‐associated AP‐1 controls termination of STING signalling. Nature. 2022;610(7933):761‐767. doi:10.1038/s41586-022-05354-0 36261523 PMC9605868

[31] Álvarez J , Fernández Real JM , Guarner F , et al. Gut microbes and health. Microbiota intestinal y salud. Gastroenterol Hepatol. 2021;44(7):519‐535. doi:10.1016/j.gastrohep.2021.01.009 33652061

[32] Frangoul H , Altshuler D , Cappellini MD , et al. CRISPR‐Cas9 gene editing for sickle cell disease and β‐thalassemia. N Engl J Med. 2021;384(3):252‐260. doi:10.1056/NEJMoa2031054 33283989

[33] Sélénou C , Brioude F , Giabicani E , Sobrier ML , Netchine I . IGF2: development, genetic and epigenetic abnormalities. Cells. 2022;11(12):1886. doi:10.3390/cells11121886 35741015 PMC9221339

[34] Zuo Q , Jin K , Wang Y , Song J , Zhang Y , Li B . CRISPR/Cas9‐mediated deletion of C1EIS inhibits chicken embryonic stem cell differentiation into male germ cells (Gallus gallus). J Cell Biochem. 2017;118(8):2380‐2386. doi:10.1002/jcb.25900 28106276

[35] Kafaja S , Valera I , Divekar AA , et al. pDCs in lung and skin fibrosis in a bleomycin‐induced model and patients with systemic sclerosis. JCI Insight. 2018;3(9):e98380. doi:10.1172/jci.insight.98380 29720568 PMC6012518

[36] Mahmoudi S , Mancini E , Xu L , et al. Heterogeneity in old fibroblasts is linked to variability in reprogramming and wound healing. Nature. 2019;574(7779):553‐558. doi:10.1038/s41586-019-1658-5 31645721 PMC7253295

[37] Yang P , Luo Q , Wang X , et al. Comprehensive analysis of fibroblast activation protein expression in interstitial lung diseases. Am J Respir Crit Care Med. 2023;207(2):160‐172. doi:10.1164/rccm.202110-2414OC 35984444 PMC9893314

[38] Ma L , Hernandez MO , Zhao Y , et al. Tumor cell biodiversity drives microenvironmental reprogramming in liver cancer. Cancer Cell. 2019;36(4):418‐430. doi:10.1016/j.ccell.2019.08.007 e6 31588021 PMC6801104

[39] Butler A , Hoffman P , Smibert P , Papalexi E , Satija R . Integrating single‐cell transcriptomic data across different conditions, technologies, and species. Nat Biotechnol. 2018;36(5):411‐420. doi:10.1038/nbt.4096 29608179 PMC6700744

[40] Hong M , Tao S , Zhang L , et al. RNA sequencing: new technologies and applications in cancer research. J Hematol Oncol. 2020;13(1):166. doi:10.1186/s13045-020-01005-x 33276803 PMC7716291

[41] Wu Y , Liu X , Li G . Integrated bioinformatics and network pharmacology to identify the therapeutic target and molecular mechanisms of Huangqin decoction on ulcerative Colitis. Sci Rep. 2022;12(1):159. doi:10.1038/s41598-021-03980-8 34997010 PMC8741777

[42] Yun TJ , Igarashi S , Zhao H , et al. Human plasmacytoid dendritic cells mount a distinct antiviral response to virus‐infected cells. Sci Immunol. 2021;6(58):eabc7302. doi:10.1126/sciimmunol.abc7302 33811059 PMC8221820

[43] Lippens C , Duraes FV , Dubrot J , et al. IDO‐orchestrated crosstalk between pDCs and Tregs inhibits autoimmunity. J Autoimmun. 2016;75:39‐49. doi:10.1016/j.jaut.2016.07.004 27470005 PMC5127883

[44] Zhi L , Zhao L , Zhang X , et al. SLCO1B3 promotes colorectal cancer tumorigenesis and metastasis through STAT3. Aging (Albany NY). 2021;13(18):22164‐22175. doi:10.18632/aging.203502 34526411 PMC8507254

[45] Gao X , Bao W , Bai J , Fan K , Li L , Li Y . UHMK1 aids colorectal cancer cell proliferation and chemoresistance through augmenting IL‐6/STAT3 signaling. Cell Death Dis. 2022;13(5):424. doi:10.1038/s41419-022-04877-8 35501324 PMC9061793

[46] Wang J , Zhang Y , Song H , et al. The circular RNA circSPARC enhances the migration and proliferation of colorectal cancer by regulating the JAK/STAT pathway. Mol Cancer. 2021;20(1):81. doi:10.1186/s12943-021-01375-x 34074294 PMC8167978

[47] Chim SM , Qin A , Tickner J , et al. EGFL6 promotes endothelial cell migration and angiogenesis through the activation of extracellular signal‐regulated kinase. J Biol Chem. 2011;286(25):22035‐22046. doi:10.1074/jbc.M110.187633 21531721 PMC3121348

[48] Chen S , Morine Y , Tokuda K , et al. Cancer‑associated fibroblast‑induced M2‑polarized macrophages promote hepatocellular carcinoma progression via the plasminogen activator inhibitor‑1 pathway. Int J Oncol. 2021;59(2):59. doi:10.3892/ijo.2021.5239 34195849 PMC8253588

[49] Ma JD , Jing J , Wang JW , et al. A novel function of artesunate on inhibiting migration and invasion of fibroblast‐like synoviocytes from rheumatoid arthritis patients. Arthritis Res Ther. 2019;21(1):153. doi:10.1186/s13075-019-1935-6 31234900 PMC6591920

[50] Meng M , Tan J , Chen W , et al. The fibrosis and immunological features of hypochlorous acid induced mouse model of systemic sclerosis. Front Immunol. 2019;10:1861. doi:10.3389/fimmu.2019.01861 31481954 PMC6710365

[51] Wang Y , Zhang L , Wu GR , et al. MBD2 serves as a viable target against pulmonary fibrosis by inhibiting macrophage M2 program. Sci Adv. 2021;7(1):eabb6075. doi:10.1126/sciadv.abb6075 33277324 PMC7775789

[52] Wang H , Xu H , Lyu W , et al. KLF4 regulates TERT expression in alveolar epithelial cells in pulmonary fibrosis. Cell Death Dis. 2022;13(5):435. doi:10.1038/s41419-022-04886-7 35508454 PMC9068714

[53] Dorst DN , van Caam APM , Vitters EL , et al. Fibroblast activation protein targeted photodynamic therapy selectively kills activated skin fibroblasts from systemic sclerosis patients and prevents tissue contraction. Int J Mol Sci. 2021;22(23):12681. doi:10.3390/ijms222312681 34884484 PMC8657852

[54] Liu X , Zhang P , Zhang X , et al. Fgf21 knockout mice generated using CRISPR/Cas9 reveal genetic alterations that may affect hair growth. Gene. 2020;733:144242. doi:10.1016/j.gene.2019.144242 31743770

[55] Xu M , Xu H , Chen J , Chen C , Xu F , Qin Z . Generation of conditional Acvrl1 knockout mice by CRISPR/Cas9‐mediated gene targeting. Mol Cell Probes. 2018;37:32‐38. doi:10.1016/j.mcp.2017.11.003 29129659

[56] Yan Y , Ding X , Li K , et al. CNS‐specific therapy for ongoing EAE by silencing IL‐17 pathway in astrocytes [published correction appears in Mol Ther. 2014 Dec;22(12):2155]. Mol Ther. 2012;20(7):1338‐1348. doi:10.1038/mt.2012.12 22434134 PMC3392982

[57] Park MJ , Park Y , Choi JW , et al. Establishment of a humanized animal model of systemic sclerosis in which T helper‐17 cells from patients with systemic sclerosis infiltrate and cause fibrosis in the lungs and skin. Exp Mol Med. 2022;54(9):1577‐1585. doi:10.1038/s12276-022-00860-7 36175484 PMC9534900

[58] Xie B , Lu C , Chen C , Zhou J , Deng Z . miR‐135a alleviates silica‐induced pulmonary fibrosis by targeting NF‐κB/inflammatory signaling pathway. Mediators Inflamm. 2020;2020:1231243. doi:10.1155/2020/1231243 32617074 PMC7317310

[59] Rieppo L , Janssen L , Rahunen K , Lehenkari P , Finnilä MAJ , Saarakkala S . Histochemical quantification of collagen content in articular cartilage. PLoS One. 2019;14(11):e0224839. doi:10.1371/journal.pone.0224839 31697756 PMC6837441

[60] Zhang QF , Li J , Jiang K , et al. CDK4/6 inhibition promotes immune infiltration in ovarian cancer and synergizes with PD‐1 blockade in a B cell‐dependent manner. Theranostics. 2020;10(23):10619‐10633. doi:10.7150/thno.44871 32929370 PMC7482823

[61] Bodewes ILA , Huijser E , van Helden‐Meeuwsen CG , et al. TBK1: a key regulator and potential treatment target for interferon positive Sjögren's syndrome, systemic lupus erythematosus and systemic sclerosis. J Autoimmun. 2018;91:97‐102. doi:10.1016/j.jaut.2018.02.001 29673738

[62] Swiecki M , Colonna M . The multifaceted biology of plasmacytoid dendritic cells. Nat Rev Immunol. 2015;15(8):471‐485. doi:10.1038/nri3865 26160613 PMC4808588

[63] Barrat FJ , Su L . A pathogenic role of plasmacytoid dendritic cells in autoimmunity and chronic viral infection. J Exp Med. 2019;216(9):1974‐1985. doi:10.1084/jem.20181359 31420375 PMC6719431

[64] Li G , Larregina AT , Domsic RT , et al. Skin‐resident effector memory CD8^+^CD28^−^ T cells exhibit a profibrotic phenotype in patients with systemic sclerosis. J Invest Dermatol. 2017;137(5):1042‐1050. doi:10.1016/j.jid.2016.11.037 28012718 PMC5433864

[65] Peters MDJ , Godfrey C , McInerney P , et al. Best practice guidance and reporting items for the development of scoping review protocols. JBI Evid Synth. 2022;20(4):953‐968. doi:10.11124/JBIES-21-00242 35102103

[66] Zhao W . Negative regulation of TBK1‐mediated antiviral immunity. FEBS Lett. 2013;587(6):542‐548. doi:10.1016/j.febslet.2013.01.052 23395611 PMC7094513

[67] He T , Xia Y , Yang J . Systemic inflammation and chronic kidney disease in a patient due to the RNASEH2B defect. Pediatr Rheumatol Online J. 2021;19(1):9. doi:10.1186/s12969-021-00497-2 33482855 PMC7821736

[68] Liu G , Sun J , Yang ZF , et al. Cancer‐associated fibroblast‐derived CXCL11 modulates hepatocellular carcinoma cell migration and tumor metastasis through the circUBAP2/miR‐4756/IFIT1/3 axis. Cell Death Dis. 2021;12(3):260. doi:10.1038/s41419-021-03545-7 33707417 PMC7952559

[69] Margaroli C , Fram T , Sharma NS , et al. Type I interferon‐dependent IFIT3 signaling is critical for viral clearance in airway neutrophils. Res Sq. 2023. doi:10.21203/rs.3.rs-1812836/v1. Preprint. rs.3.rs‐1812836. PMC10322684 37071484

[70] Wang SW , Gao C , Zheng YM , et al. Current applications and future perspective of CRISPR/Cas9 gene editing in cancer. Mol Cancer. 2022;21(1):57. doi:10.1186/s12943-022-01518-8 35189910 PMC8862238

[71] Liu C , Tang J , Luo W , et al. DNA from macrophages induces fibrosis and vasculopathy through POLR3A/STING/type I interferon axis in systemic sclerosis. Rheumatology (Oxford). 2023;62(2):934‐945. doi:10.1093/rheumatology/keac324 35686918

[72] Bai Z , Gao T , Zhang R , et al. Inhibition of IL‐6 methylation by Saikosaponin C regulates neuroinflammation to alleviate depression. Int Immunopharmacol. 2023;118:110043. doi:10.1016/j.intimp.2023.110043 36965369

