# Supplementary figures and images for: IFIT3 mediates TBK1 phosphorylation to promote activation of pDCs and exacerbate systemic sclerosis in mice
Source: Clin Transl Med. 2024 Sep 20;14(9):e1800. doi: 10.1002/ctm2.1800 (PMC11415598; doi:10.1002/ctm2.1800)

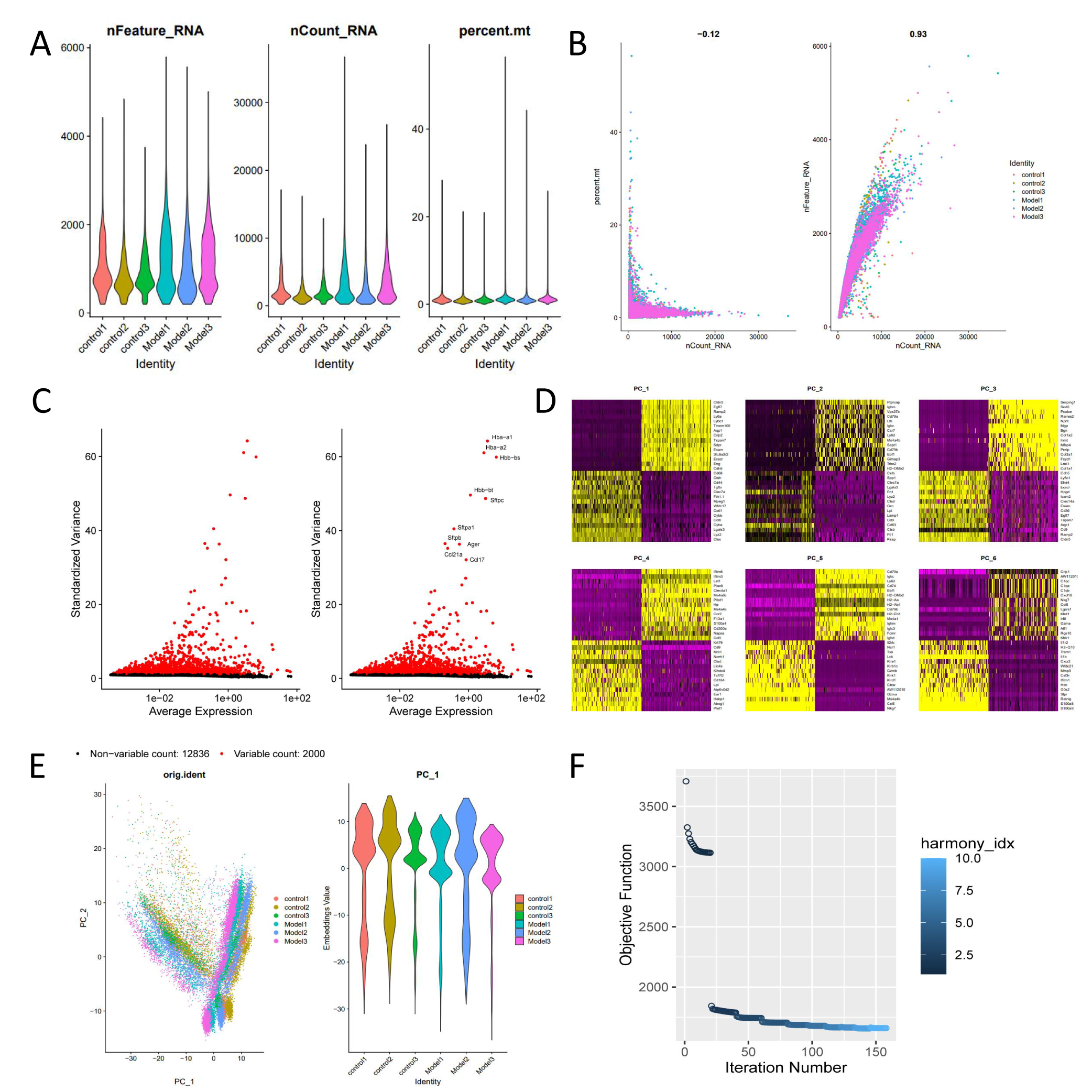

Supplement: Supplementary file 1 — Figure S1. Data quality control and PCA dimensionality reduction of scRNA‐seq. Note: (A) Violin plots showing the number of genes (nFeature_RNA), mRNA molecules (nCount_RNA), and percentage of mitochondrial genes (percent.mt) in each cell of the scRNA‐seq data. (B) Scatter plots depicting the correlation between filtered data nCount_RNA and percent.mt, as well as nCount_RNA and nFeature_RNA. (C) Differential expression analysis to identify highly variable genes, with red indicating the top 2000 highly variable genes and black representing genes with low variability. The top 10 gene names from the highly variable gene set are labelled. (D) Heatmap displaying the expression of the top 20 genes most correlated with PC_1 ‐ PC_6 in the PCA, with yellow indicating upregulation and purple indicating downregulation. (E) Distribution of cells before batch correction in PC_1 and PC_2, with each point representing a cell. (F) Batch correction process diagram using Harmony, where the x‐axis represents the number of iterations. [file CTM2-14-e1800-s002.tif]

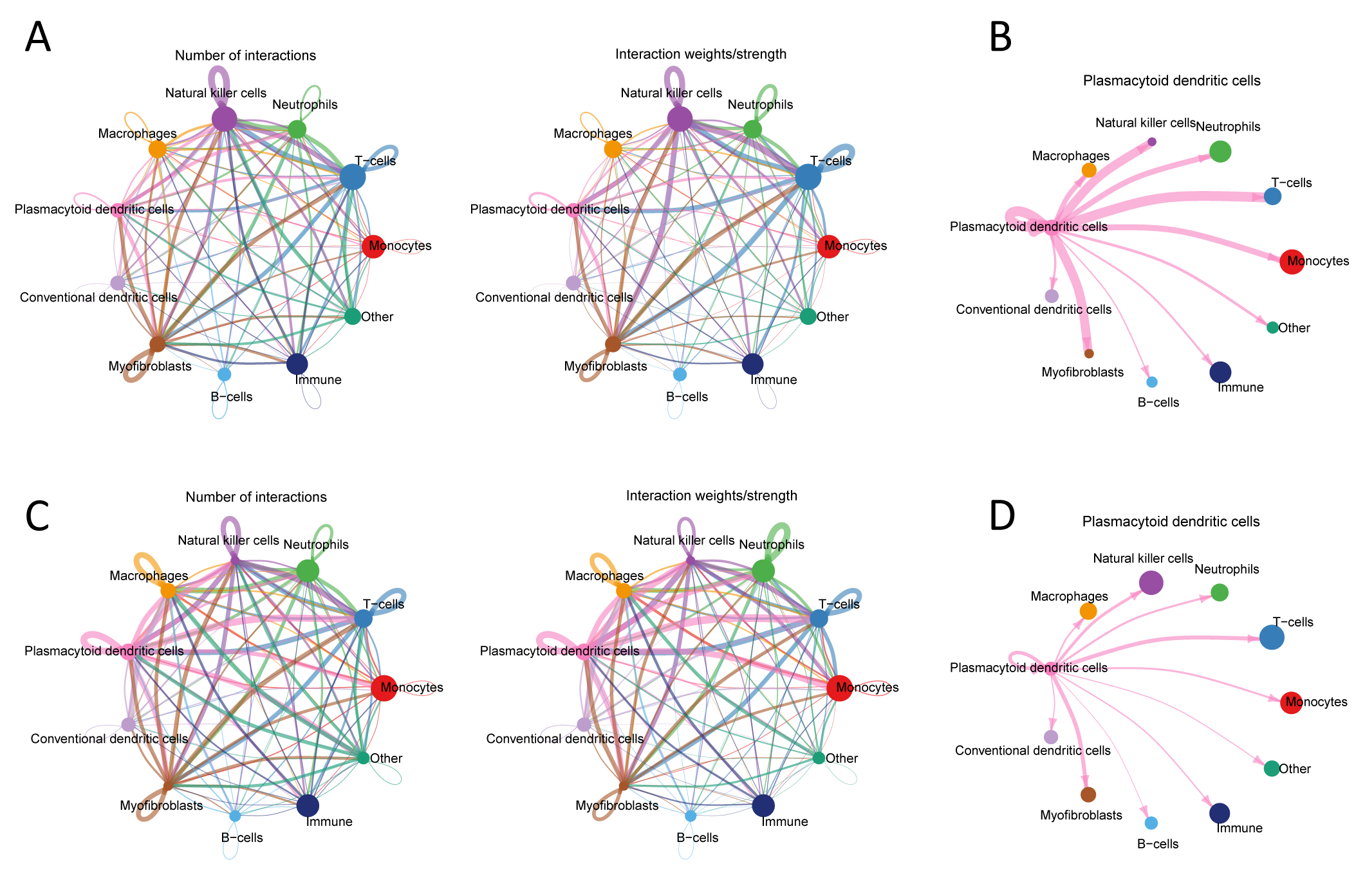

Supplement: Supplementary file 2 — Figure S2. Analysis of cell‐cell interactions in scRNA‐seq. Note: (A) Circular plot showing cell‐cell interactions in the normal group, with line thickness representing interaction strength. (B) Cell‐cell interactions of pDCs with other cells in the normal group. (C) Circular plot depicting cell‐cell interactions in the model group, with line thickness representing interaction strength. (D) Cell‐cell interactions of pDCs with other cells in the model group. [file CTM2-14-e1800-s004.tif]

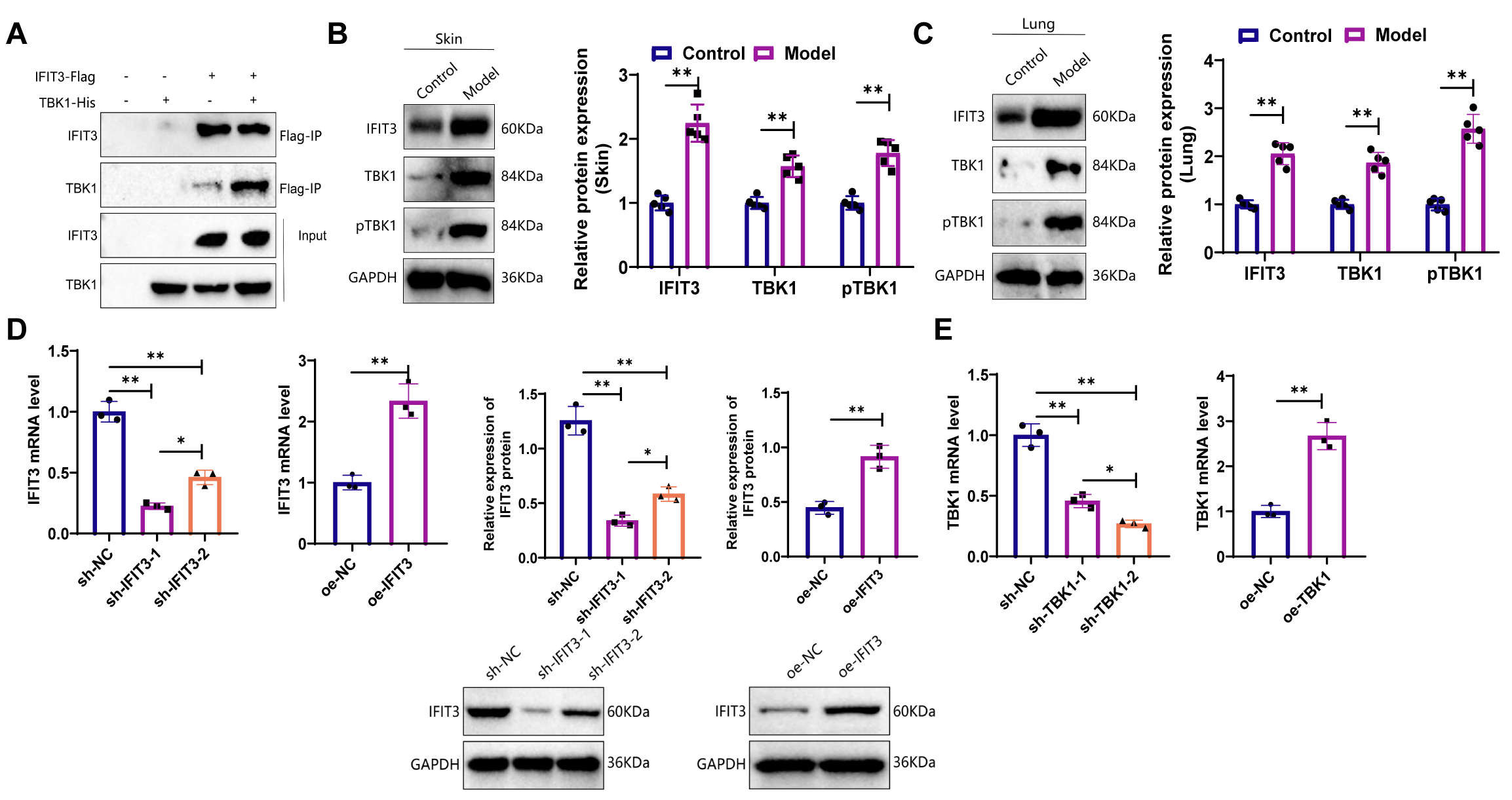

Supplement: Supplementary file 3 — Figure S3. Involvement of IFIT3 and TBK1 in the development of SSc. Note: (A) Co‐immunoprecipitation experiment of IFIT3 and TBK1; (B) Expression levels of IFIT3, TBK1, and pTBK1 in skin tissue detected by Western blot in the Bleomycin‐induced SSc mouse model. (C) Expression levels of IFIT3, TBK1, and pTBK1 in lung tissue were detected by Western blot in the Bleomycin‐induced SSc mouse model. (D) Transfection efficiency of silencing or overexpressing IFIT3 detected by RT‐qPCR and Western Blot. (E) Transfection efficiency of silencing or overexpressing TBK1 detected by RT‐qPCR. *P < 0.01, P < 0.05, animal experiments n = 5, cell experiments repeated 3 times. [file CTM2-14-e1800-s001.tif]

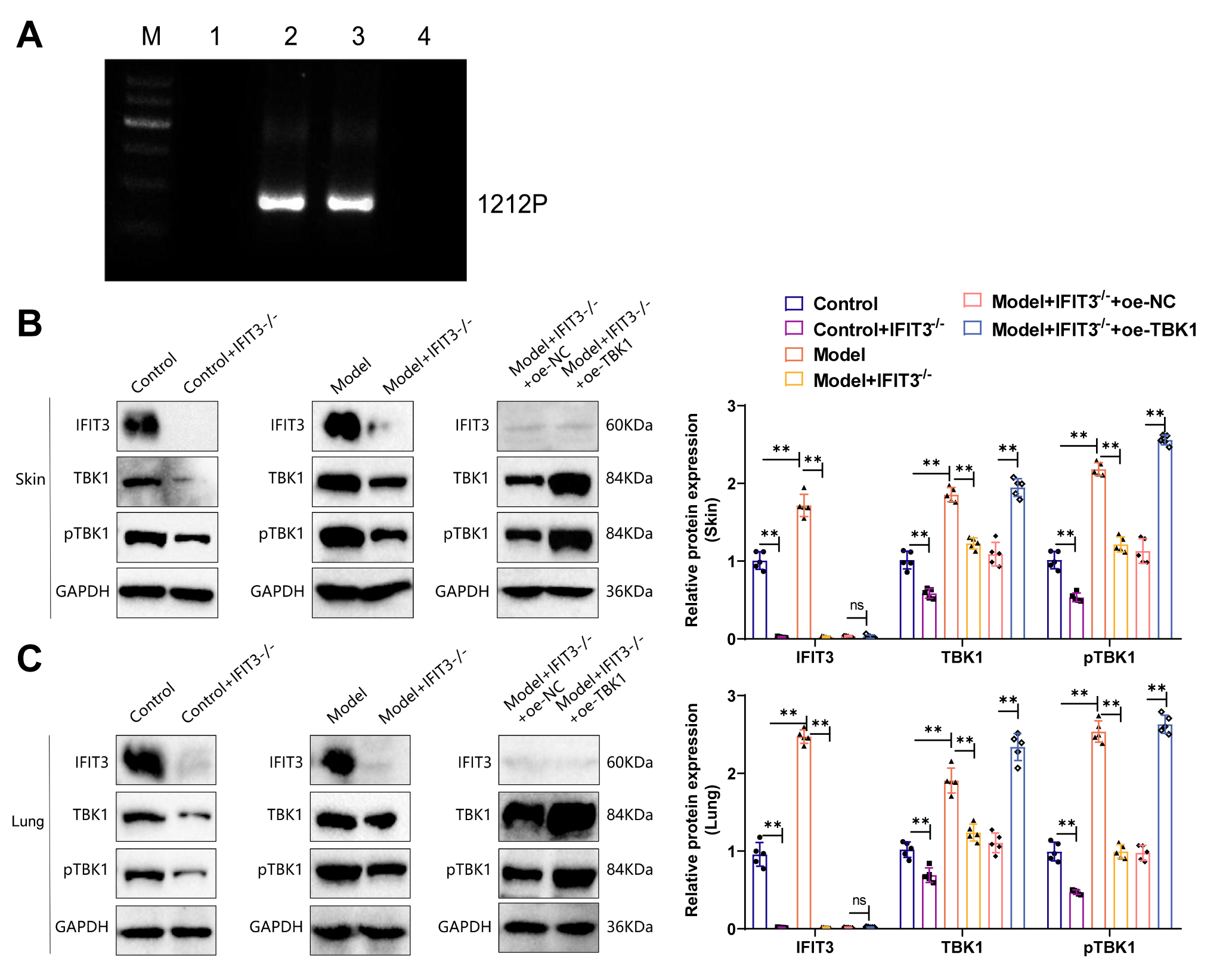

Supplement: Supplementary file 4 — Figure S4. Inhibition of TBK1 activation and release of inflammatory factors by IFIT3. Note: (A) Mouse gene typing data. M represents Marker, 1 and 4 represent IFIT3−/− mice, 2 and 3 represent wild‐type mice; (B) Expression levels of IFIT3, TBK1, and pTBK1 in skin tissue detected by Western blot. (C) Western blot detected expression levels of IFIT3, TBK1, and pTBK1 in lung tissue. **P < 0.05, animal experiments n = 5. [file CTM2-14-e1800-s003.tif]
